# Competitive solvent-molecule interactions govern primary processes of diphenylcarbene in solvent mixtures

**DOI:** 10.1038/ncomms12968

**Published:** 2016-10-06

**Authors:** Johannes Knorr, Pandian Sokkar, Sebastian Schott, Paolo Costa, Walter Thiel, Wolfram Sander, Elsa Sanchez-Garcia, Patrick Nuernberger

**Affiliations:** 1 Physikalische Chemie II, Ruhr-Universität Bochum, 44780 Bochum, Germany; 2 Max-Planck-Institut für Kohlenforschung, Kaiser-Wilhelm-Platz 1, 45470 Mülheim an der Ruhr, Germany; 3 Institut für Physikalische und Theoretische Chemie, Universität Würzburg, Am Hubland, 97074 Würzburg, Germany; 4 Organische Chemie II, Ruhr-Universität Bochum, 44780 Bochum, Germany

## Abstract

Photochemical reactions in solution often proceed via competing reaction pathways comprising intermediates that capture a solvent molecule. A disclosure of the underlying reaction mechanisms is challenging due to the rapid nature of these processes and the intricate identification of how many solvent molecules are involved. Here combining broadband femtosecond transient absorption and quantum mechanics/molecular mechanics simulations, we show for one of the most reactive species, diphenylcarbene, that the decision-maker is not the nearest solvent molecule but its neighbour. The hydrogen bonding dynamics determine which reaction channels are accessible in binary solvent mixtures at room temperature. In-depth analysis of the amount of nascent intermediates corroborates the importance of a hydrogen-bonded complex with a protic solvent molecule, in striking analogy to complexes found at cryogenic temperatures. Our results show that adjacent solvent molecules take the role of key abettors rather than bystanders for the fate of the reactive intermediate.

## Abstract (web summary)

Photochemistry in solution often involves coexisting reaction channels that may comprise intermediates capturing a solvent molecule. Here, the authors show for one of the most reactive species, diphenylcarbene, that the decision-maker is not the nearest solvent molecule but its neighbour.

## Graphical abstract

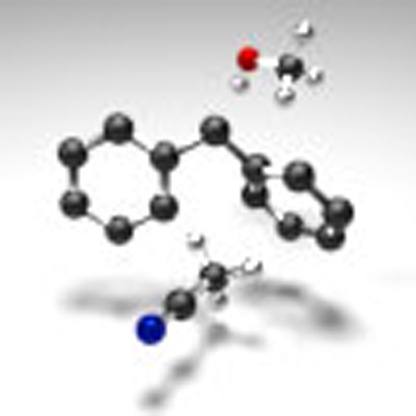

Carbene chemistry is a multifaceted research area by virtue of the highly spin-dependent reactivity of carbenes^1^. Various spectroscopic investigations ranging from laser flash-photolysis^2^,^3^,^4^,^5^,^6^,^7^,^8^,^9^,^10^,^11^,^12^, matrix-isolation studies^13^ to ultrafast measurements in different environments^14^,^15^,^16^,^17^,^18^,^19^,^20^,^21^,^22^ have unveiled the role of carbenes as intermediates in (photo)chemical reactions. In particular, the reactivity of diphenylcarbene (diphenylmethylene, Ph_2_C) intrigues researchers owing to the simplicity of the system accompanied by the variety of possible reaction pathways depending on whether the spin-configuration is either a singlet or a triplet state^23^,^24^,^25^. Singlet carbenes are assumed to insert into O–H bonds, whereas triplet carbenes insert into C–H bonds^5^. Studies by Eisenthal and coworkers^2^,^3^,^4^,^5^,^6^,^7^,^8^,^9^,^10^,^11^ have pioneered many aspects of Ph_2_C reactivity in solution, for example, effects of solvent polarity and selected cosolvents, but without the direct spectroscopic observation of the singlet ^1^Ph_2_C. This was possible in ultrafast studies, performed initially by the Chergui group^14^ and further extended by Kohler and coworkers^15^ in several pure solvents. The latter revealed that ultraviolet excitation of the diazo-compound precursor diphenyldiazomethane (Ph_2_CN_2_) leads to formation of ^1^Ph_2_C on a sub-picosecond time scale, which can further react via intersystem crossing (ISC) to the triplet ^3^Ph_2_C in a few hundreds of picoseconds in aprotic solvents. However, if ^1^Ph_2_C is allowed to react with alcohols, an ultrafast intermolecular proton-transfer can occur, leading to an intermediate benzhydryl cation (Ph_2_CH^+^), and eventually to a diphenylalkyl ether, the formal product of O–H insertion. Kirmse and Steenken^12^,^26^ inferred that two alcohol molecules are needed for the protonation. Others postulated a concerted reaction pathway, possibly involving an intermediate ylide^27^,^28^,^29^. Recent findings in matrix-isolation experiments at low temperatures provided additional aspects of diphenylcarbene reactivity: in argon matrices doped with 0.5–1% methanol (MeOH), ^1^Ph_2_C (in contrast to ^3^Ph_2_C) forms a hydrogen-bonded complex with a MeOH molecule, hence refuting the assumption of a general triplet ground state^30^. Whereas similar results have been found when replacing the MeOH dopants by water molecules, an amorphous water environment enabled the isolation of stable Ph_2_CH^+^ without the need for superacidic conditions^31^.

While the behaviour of Ph_2_C in pure solvents has been investigated, the emphasis of our study is also on the dynamics in solvent mixtures, because the possible reaction channels of ^1^Ph_2_C accessible under different conditions will compete with each other when the solvent environment is systematically varied. Here we combine hybrid quantum mechanics/molecular mechanics (QM/MM) calculations and advanced femtosecond pump-probe spectroscopy to unravel the fate of ^1^Ph_2_C in binary mixtures of the protic solvent MeOH and the aprotic acetonitrile (MeCN) at room temperature. By going from neat MeOH to pure MeCN, a tailored solvent environment with variable distances between the carbene and the alcohol molecules is created, also aimed at ‘mimicking' the MeOH doping of the matrix-isolation studies. The joint theoretical and experimental investigation directly visualizes the interplay and competition of the solvent molecules in the mixtures, discloses the amount of solvent molecules necessary for certain reaction pathways and allows the identification of a concerted reaction pathway that involves an intermediate complex ^1^Ph_2_C^…^HOMe. Our results emphasize that ultrafast photodynamics in solvent mixtures are more than just a linear combination of the behaviour in neat solvents, and that the amount of molecules of a certain solvent in the vicinity of the solute may be of critical importance for a desired reaction outcome.

## Results

### Computational studies

The following experimental and theoretical studies aim at understanding the competition between reaction channels accessible not only in pure solvents, but also in solvent mixtures on an ultrafast time scale. For a mechanistic understanding at the molecular level, hybrid QM/MM calculations were employed to capture the solvent effects and the specific interactions governing the stability of Ph_2_C (see Methods and Supplementary Table 1). We performed solvent-only simulations to validate the behaviour of the physical properties of pure MeOH, pure MeCN and a mixture of MeCN:MeOH (80:20% v/v) during the simulations (Supplementary Table 1 and Supplementary Note 1). The simulations confirm that the solvent structure involves hydrogen-bonds in pure MeOH and that there is no well-structured solvent arrangement in pure MeCN (Supplementary Fig. 1a and Supplementary Fig. 1b). For the mixture, the simulations indicate the absence of phase separation or aggregation, which is in accordance with experimental observations (see Supplementary Fig. 1c, Supplementary Fig. 2 and Supplementary Note 2) of rather weak preferential solvation^32^.

**Figure 1:**
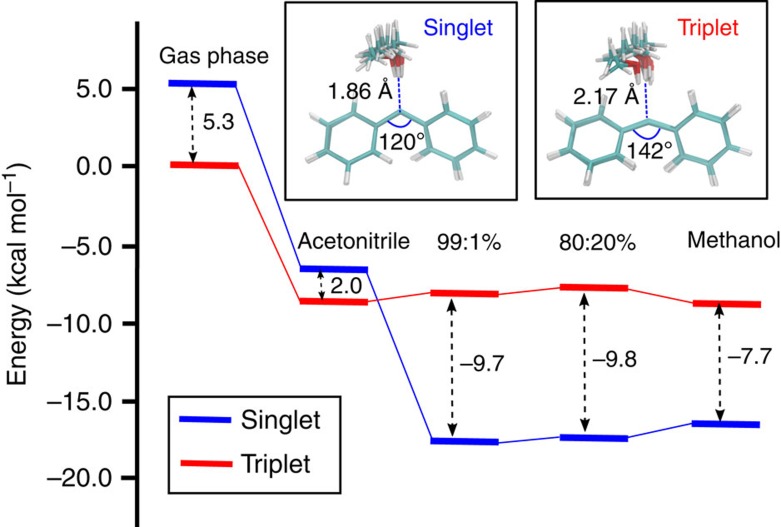
Calculated singlet-triplet energy gaps of Ph_2_C. The values (kcal mol^−1^) correspond to Ph_2_C in the gas phase, pure solvents and different mixtures of MeOH and MeCN. The experimental gap of Ph_2_C in acetonitrile is reported as 2.6 kcal mol^−1^ by Eisenthal *et al*.^5^ The insets show the H-bonded complexes of MeOH with ^1^Ph_2_C and ^3^Ph_2_C.

In order to elucidate whether a solvent molecule can significantly influence the balance of the Ph_2_C spin states, we analysed the average QM energies of ^1^Ph_2_C and ^3^Ph_2_C in explicit solvent (QM/MM calculations, see Supplementary Figs 3 and 4 and Supplementary Note 3). Although pure MeCN stabilizes the singlet more than the triplet, the latter is energetically still favoured, as in the gas phase. The situation already changes by addition of small amounts of MeOH, reversing the singlet-triplet energy gap (see Fig. 1 and Supplementary Tables 2–4). The optimized structures reveal that MeOH forms a strong H-bond with ^1^Ph_2_C, thereby lowering its energy, whereas in the case of the less polar ^3^Ph_2_C the stabilization is much less pronounced. This behaviour is also directly reflected in the average distance of the H-bond in the complex ^1^Ph_2_C^…^HOMe (inset in Fig. 1). Remarkably, the lowest energy for ^1^Ph_2_C is not found in neat MeOH, but at very low concentrations of MeOH. This suggests that ^1^Ph_2_C is less stabilized if the MeOH molecule interacting with the carbene centre is also involved in an H-bonding network with other neighbouring methanol molecules. A series of QM/MM molecular dynamics (MD) simulations also corroborated that ^1^Ph_2_C forms a hydrogen bonded complex in pure MeOH, while ^3^Ph_2_C does not (Supplementary Fig. 5). Such complexes between ^1^Ph_2_C and MeOH are also observed in solvent mixtures with lower MeOH content, but the complex formation strongly depends on the diffusion of methanol molecules to approach the carbene centre (Supplementary Figs 6 and 7 and Supplementary Note 4).

**Figure 2:**
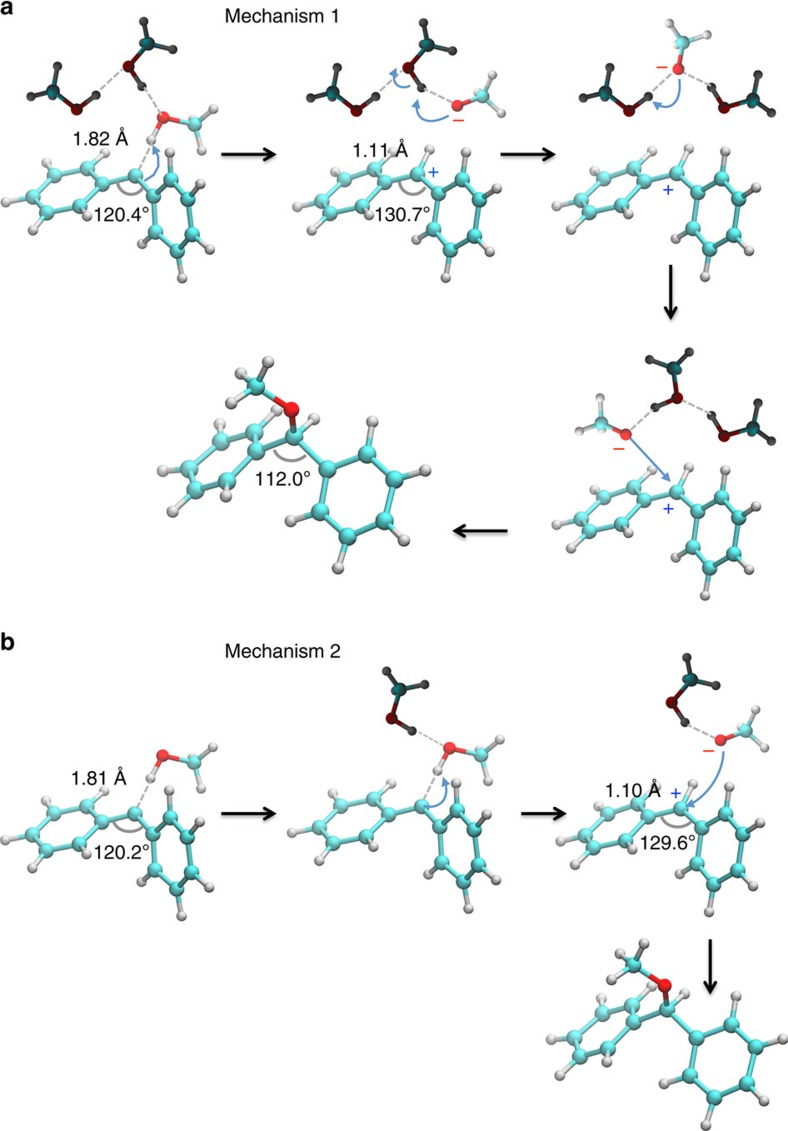
Mechanisms 1 and 2 of O–H insertion for the reaction of ^1^Ph_2_C with methanol. (**a**) Mechanism 1, (**b**) Mechanism 2. Average OḢ̇̇C_1Ph2C_ distances and CCC_1Ph2C_ angles are shown.

On an ultrafast time scale, ^1^Ph_2_C reacts with an adjacent MeOH molecule. Further simulations unveil that the reaction leading to O–H insertion always starts with the protonation of ^1^Ph_2_C, but two different mechanisms are found. In mechanism 1 (Fig. 2a), H-bond formation with a MeOH molecule is almost instantly followed by transfer of a proton to the carbene to yield Ph_2_CH^+^, while the nascent methoxide ion takes up a proton from a neighbouring MeOH molecule. In this way, the two charges are spatially separated, and the initially H-bonded MeOH molecule is not the one found in the final ether product. Instead, a different MeOH molecule later on transfers a proton to the methoxide ion, and combination of this newly formed methoxide with Ph_2_CH^+^ results in the formation of the ether. In mechanism 2, the proton transfer occurs within the ^1^Ph_2_C^…^HOMe complex, and the resulting methoxide anion combines with Ph_2_CH^+^ to the final product. The protonation step is facilitated by a second MeOH molecule, which does not directly interact with the carbene (Fig. 2b). From the ten computed trajectories corresponding to ^1^Ph_2_C in pure MeOH, four followed mechanism 1 and five followed mechanism 2. One trajectory resulted in the formation of a side product (addition of methoxide to the phenyl ring, forming the σ-complex of a nucleophilic aromatic substitution reaction).

Concerning mechanism 2, it is worth mentioning that after the protonation step, a reversible proton transfer from the neighbouring MeOH to the methanol in the ^1^Ph_2_C^…^HOMe complex was observed in one of the five trajectories. Unlike in the other four trajectories, where Ph_2_CH^+^ instantaneously reacted with the methoxide anion to form the insertion product, this reversible proton transfer contributed to stabilize the benzhydryl cation.

It should also be noted that in neat methanol, the second solvent molecule depicted in mechanism 2 is found already interacting with the methanol molecule hydrogen bonded to ^1^Ph_2_C. On the other hand, this is not necessarily the case in the solvent mixtures. There, it also happens that the second methanol diffuses after hydrogen bond formation towards the ^1^Ph_2_C^…^HOMe complex to facilitate the protonation reaction (Fig. 2b). In both cases the protonation reaction does not occur without a second MeOH molecule in the vicinity (see Supplementary Fig. 8 and Supplementary Note 5). This observation clearly suggests that a pathway involving the reaction of ^1^Ph_2_C with a single H-bonded MeOH molecule is disfavoured.

### Experimental studies

**Figure 3:**
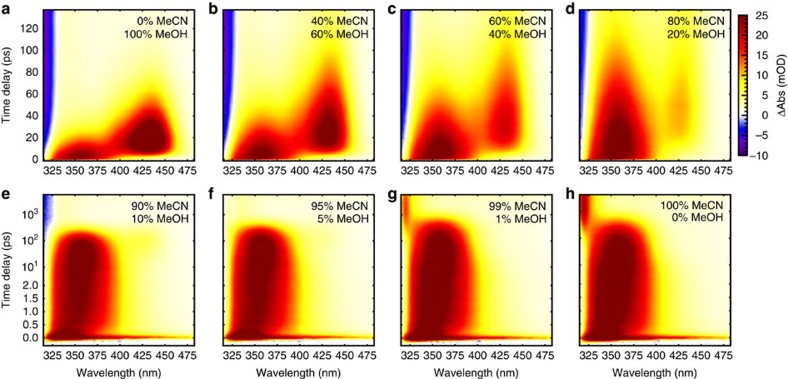
Transient absorption of Ph_2_CN_2_ under 285 nm excitation. Note that for concentrations up to 80% MeCN (**a**–**d**), data is shown using a linear time axis covering the first 135 ps pump-probe delay, whereas for higher MeCN concentrations (**e**–**h**) a lin-log time axis is chosen in order to include dynamics up to 4 ns pump-probe delay.

Complementing our simulations, broadband transient absorption (TA) measurements on Ph_2_CN_2_ in solvent mixtures of MeOH and MeCN were conducted (see Methods) and intermediate species were identified by characteristic absorption signals. An extract of these measurements is presented in Fig. 3, a movie of all measurements is available as Supplementary Information. In the case of neat methanol (top left panel), two distinct positive TA signals are observed, which correspond to the absorption of ^1^Ph_2_C centred at 355 nm and of the benzhydryl cation Ph_2_CH^+^ at 435 nm, respectively. The ground state bleach of the precursor Ph_2_CN_2_ appears on the high-energy edge of the detection range as negative TA signal. The spectral positions agree both with Kohler's study^15^ and with reported matrix experiments^30^,^31^ for Ph_2_CH^+^. Furthermore, they agree with recent measurements of the Riedle group on the photoinitiated carbocation formation from benzhydryl chlorides^33^,^34^,^35^. Additionally, and independent of the solvent mixing ratio, we observe the spectral signature of the excited diazo compound Ph_2_CN_2_* at around 335 nm (Supplementary Fig. 9 and Supplementary Note 6), which decays with a time constant of 150 fs. For MeCN fractions above 90%, the ground state bleach signal is completely overlaid by the positive TA contributions of the reaction intermediates.

**Figure 4:**
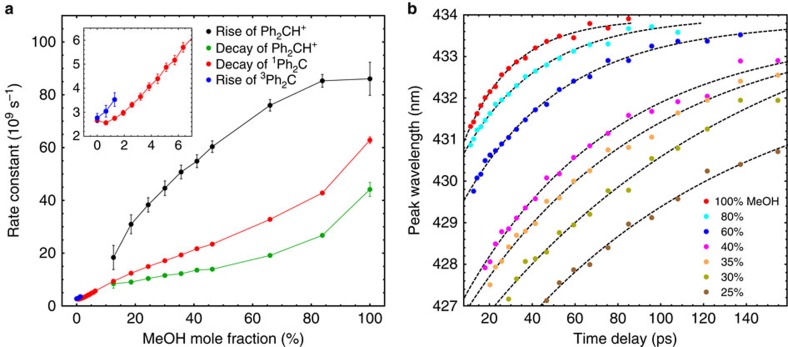
Effects of solvent mixing ratio on reaction dynamics. (**a**) Rate constants extracted from nonlinear interpolation describing the rise and the decay of Ph_2_CH^+^ (maximum wavelength), the decay of ^1^Ph_2_C (355 nm), as well as the rise of ^3^Ph_2_C (315 nm) in solvent mixtures of varying MeOH mole fractions. The bars indicate 95% confidence intervals (±1.96 s.d.). (**b**) Time-dependent peak position of the Ph_2_CH^+^ absorption band. For all solvent mixtures, a red-shift is observed, but the initial wavelength increases with MeOH volume fraction. See Supplementary Tables 6–9 for the data, Supplementary Figs 11–13 and Supplementary Note 6 for details on analysis and fitting.

The competition between different reaction pathways becomes evident from the TA data in solvent mixtures, where an increase in MeCN concentration leads to several changes in the dynamics: (1) the characteristic time scales increase, so ^1^Ph_2_C decays more slowly, while Ph_2_CH^+^ is formed later and survives for a longer time (see Fig. 3 and rate constants displayed in Fig. 4a). This observation confirms that reactions of the initially formed ^1^Ph_2_C depend on the MeOH concentration, and so does the formation and decay of Ph_2_CH^+^; (2) the decay rate of ^1^Ph_2_C is always lower than the rate describing the rise of Ph_2_CH^+^. Hence, the absorption centred at 355 nm cannot originate solely from a precursor of Ph_2_CH^+^, but must also be due to a species which can co-exist with Ph_2_CH^+^ in solution and which has a similar electronic absorption as ^1^Ph_2_C. We, therefore, conclude that the TA signal at 355 nm stems both from ^1^Ph_2_C and the complex ^1^Ph_2_C^…^HOMe. This conclusion is supported by our NEVPT2/CASSCF calculations of the ultraviolet–visible transitions of ^1^Ph_2_C and its complexes with water and MeOH (see Supplementary Fig. 10, Supplementary Table 5, and Supplementary Note 7 for more details); (3) the Ph_2_CH^+^ absorption becomes weaker compared to the initial ^1^Ph_2_C signal (see for example, the panel for 80% MeCN in Fig. 3). This directly shows that for reduced MeOH concentrations, Ph_2_CH^+^ is less likely to be formed compared with ^1^Ph_2_C^…^HOMe; (4) for low MeOH fractions, the Ph_2_CH^+^ signal disappears and a new absorption around 315 nm is observed after several hundred picoseconds (lower panels in Fig. 3). At very low concentrations of MeOH, the ISC pathway to ^3^Ph_2_C absorbing at 315 nm^15^,^25^ becomes dominant.

Additional information on solvation effects is deduced from the ultrafast dynamics in the solvent mixtures. Figure 4b displays the temporal evolution of the maximum absorption wavelength of the Ph_2_CH^+^ intermediate as derived from the TA data. For all mixtures with a sufficient amount of Ph_2_CH^+^, the starting position is at lower wavelengths for lower MeOH fractions, as a consequence of the solvent polarity^36^ and the degree of solvation of the precursor ^1^Ph_2_C in the different mixtures. Furthermore, a red-shift of the Ph_2_CH^+^ signal is observed, which originates from initial solvation of the cation, but after several picoseconds mostly reflects intramolecular geometrical changes and the separation of the benzhydryl cation and the methoxide anion. This separation leads to a shrinking energy gap between the ground and excited states of the benzhydryl cation, as shown by Riedle and coworkers in studies on benzhydryl halides^35^. Hence, the simultaneously generated methoxide anion does not geminately react with Ph_2_CH^+^, which thereby becomes observable in the experiments. While diffusion may contribute, we propose that the separation process involves a second MeOH molecule, as suggested in refs 12, 26 and also confirmed by our simulations. For the singlet carbene absorption (see Supplementary Fig. 14 and Supplementary Note 6), we observe a red-shift during the first ten ps. The solvent environment adjusted to the diazo precursor has to rearrange upon the formation of ^1^Ph_2_C, and this solvent rearrangement is reflected in the spectral shift which for carbenes is bathochromic^17^.

**Figure 5:**
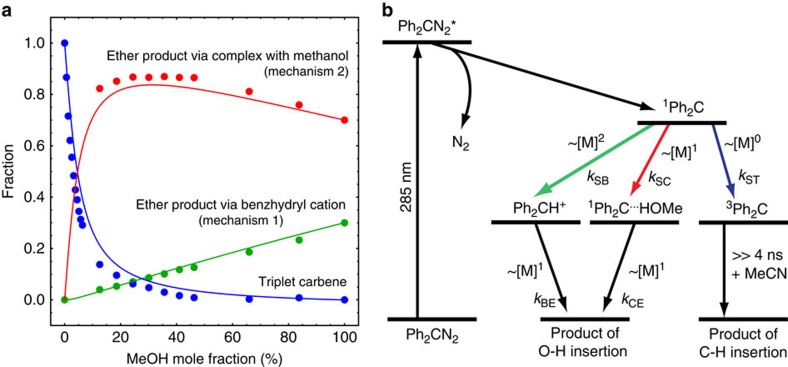
Analysis of reaction channels. (**a**) Fraction of molecules following the Ph_2_CH^+^ (green), the ^3^Ph_2_C (blue) and the ^1^Ph_2_C^…^HOMe (red) pathway for different MeOH/MeCN solvent mixtures, as derived from the TA data. The solid curves result from the reaction scheme sketched in **b**, where the rates exhibit different dependencies on the MeOH concentration [M].

The experiments described above allow us to estimate the amount of molecules following the different reaction pathways. Starting out with the dynamics in neat MeOH, a fraction *x* of the initial ^1^Ph_2_C molecules takes up a proton to form Ph_2_CH^+^. This charged species then rapidly reacts on to an ether which is dark (that is, non-absorbing) in the accessible spectral range. Kohler and coworkers^15^ showed that about 30% of the photoexcited Ph_2_CN_2_ molecules lead to Ph_2_CH^+^, but an assignment of whether the other 70% do not lead to the formation of ^1^Ph_2_C (that is, *x*=1) or follow an alternative reaction pathway to the dark product (that is, *x*=0.3) could not be made. We conclude the latter scenario occurs as for all mixtures and even in neat MeOH (Fig. 4a) the decay rate for the singlet carbene is lower than the rise of Ph_2_CH^+^, corroborating ^1^Ph_2_C^…^HOMe formation. Hence, *x* must be smaller than one but in principle could also be larger than 0.3. A direct comparison of the TA data for different mixtures is possible after normalization to the initial ^1^Ph_2_C signal and taking into account the two extreme cases, that is, that in neat MeOH 30% react via Ph_2_CH^+^, whereas in pure MeCN 100% form ^3^Ph_2_C. The latter assumption is evidenced by the matching decay rate of ^1^Ph_2_C and rise rate of ^3^Ph_2_C in Fig. 4a and the absence of other singlet decay channels in pure MeCN (ref. 15). The estimated fractions of molecules following the benzhydryl cation and the triplet pathways are displayed in Fig. 5a; the derivation of these results from the TA data is given in the Supplementary Note 8 and Supplementary Table 10. The fact that both fractions do not add up to one (independent of the value *x*) confirms the existence of a third channel (occurring more frequently in solvent mixtures) attributed to ^1^Ph_2_C^…^HOMe. The red data points in Fig. 5a correspond to the amount of molecules following this pathway, as indirectly deduced from the other fractions.

The results from the TA experiments allow us to construct a reasonable reaction scheme, which furthermore is in full accordance with the simulated mechanisms and previous studies in cryogenic matrices^30^. The separation of cation and anion indicates that more than one MeOH molecule is involved in the Ph_2_CH^+^ formation. This is included in the scheme as the simple case of a quadratic dependence on the MeOH concentration. Furthermore, the pathway via the complex ^1^Ph_2_C^…^HOMe was found, which depends linearly on the MeOH concentration. Both of these reactions lead to the ether as final product, but for the final step a further molecule of MeOH is required. The third pathway is the ISC to ^3^Ph_2_C, which contributes more at lower MeOH fractions. A reaction scheme comprising all of these aspects is sketched in Fig. 5b. An analytical solution describing the associated dynamics is given in the Supplementary Note 9 and Supplementary Figs 15–17, together with a discussion of why simpler models cannot reliably describe the experimental data.

To model the product channels shown in Fig. 5 using this reaction scheme, the corresponding three rate constants connected to the depopulation of ^1^Ph_2_C are required. The ISC rate constant can be calculated from literature data by combining the polarity dependence of this rate^11^ with the polarity of the solvent mixtures^36^. The other two are estimated from the experimental decay rate of ^1^Ph_2_C in pure MeOH (see Supplementary Note 6 for fitting procedure) and the assumption *x*=0.3. Note that no other parameter, in particular no free parameter is needed. This reaction scheme accurately describes the experimental data (solid lines in Fig. 5a), and confirms that there is a solvent mixing ratio for which the amount of molecules reacting via the ^1^Ph_2_C^…^HOMe complex reaches a maximum.

Eisenthal and coworkers^8^,^9^ found by comparing product ratios that, for ^1^Ph_2_C in aprotic solvents with small amounts of MeOH cosolvent, the alcohol exerts a ‘twofold effect': on the one hand stabilizing the singlet carbene and on the other hand opening a decay channel via the concerted O-H insertion. Our TA data discloses this behavior in a straightforward way via the decay rate of the singlet carbene: the lowest value is not observed for pure MeCN but around 99.5% (inset of Fig. 4a), directly confirming that single MeOH molecules have a stabilizing effect on ^1^Ph_2_C. Beyond that, these room-temperature studies in solution nicely complement the argon matrix experiments at 3 K doped with 1% MeOH in which also the ^1^Ph_2_C^…^HOMe complex is formed by reaction of Ph_2_C with single molecules of MeOH, eventually yielding the ether product in a tunnelling process^30^.

## Discussion

Our combined experimental and theoretical results provide a clear and conclusive picture of the fate of ^1^Ph_2_C formed photochemically in solution, thereby considering three competing reaction channels with different dependencies on the MeOH concentration. On the one hand, at low MeOH concentrations, ^1^Ph_2_C can form an H-bonded complex with a MeOH molecule. Subsequently, after a second MeOH molecule approaches, the ether product can be formed according to mechanism 2. Since the same MeOH molecule transfers the proton and completes the reaction, signatures of charged intermediates are extremely short-lived, if observable at all. Unlike in the solvent mixtures, in pure methanol a second solvent molecule can be easily found interacting with the hydrogen-bonded methanol. Our analysis suggests that mechanism 2 is the dominant mechanism in neat MeOH (see Fig. 5). On the other hand, as ^1^Ph_2_C encounters a MeOH molecule that already interacts with one (or more) neighbouring MeOH, mechanisms 1 or 2 can take place. Due to the separation of Ph_2_CH^+^ and the methoxide, the charged species produced by mechanism 1 survive for a longer time, and Ph_2_CH^+^ is identifiable by its characteristic absorption.

Concluding, our results permit two main inferences: singlet diphenylcarbene ^1^Ph_2_C, formed on a femtosecond time scale in binary solvent mixtures (MeOH/MeCN), is subject to three reaction mechanisms depending on the number of MeOH molecules that this reactive species initially encounters: (i) ISC to ^3^Ph_2_C, (ii) formation of an H-bonded complex ^1^Ph_2_C^…^HOMe, which reacts on to the ether product in a concerted fashion activated by a further MeOH molecule or (iii) ether production via an intermediate Ph_2_CH^+^, which requires that two MeOH molecules interact concurrently with ^1^Ph_2_C. Additionally, the concentration of ‘available' molecules of MeOH, that is, molecules that can hydrogen bond with ^1^Ph_2_C or facilitate the reaction via MeOH–MeOH interactions, rather than the absolute concentration of MeOH in MeCN solutions, determines the reaction pathway, which is in striking analogy with results from matrix isolation experiments at cryogenic temperatures^30^. Hence, variation of the solvent mixing ratio provides a means not only to control the time scales of the reaction, but also the reactivity, that is, which reaction path is followed.

In more general terms, even if an intermediate in solution may formally just react with a single solvent molecule, further, not directly involved solvent molecules may tip the scales in favour of a certain reaction mechanism. This nonlinear behaviour is not limited to binary solvent mixtures, and is of general importance for all intermediates capable of reacting with solvents. Our study demonstrates that solvent mixtures, if understood correctly, are versatile tools to control chemical reactivity.

## Methods

### Simulations

The reactivity of diphenylcarbene (Ph_2_C) and the relative stabilities of its singlet (^1^Ph_2_C) and triplet (^3^Ph_2_C) states in different solvent conditions, (I) acetonitrile (MeCN), (II) methanol (MeOH), (III) MeCN/MeOH (80:20% v/v mixture) and (IV) MeCN/MeOH (99:1% v/v mixture) were investigated using QM/MM calculations to capture the solvent effects and specific interactions regulating the chemistry of Ph_2_C.

### Force field parameterization

Classical force field parameters for MeOH and MeCN are available in the OPLS all-atom force field, which is able to reproduce the physical properties of these solvents in liquid simulations^37^. Since, currently, the OPLS force field is not supported by the ChemShell code^38^,^39^, the OPLS parameters of MeOH and MeCN were fitted to the CHARMM force field format. OPLS parameterization requires scaling-down of 1–4 non-bonded interactions (both van der Waals and Coulomb interactions) by 50%, in contrast to full scaling in CHARMM. The non-bonded interaction, bond stretching and bond-angle bending parameters were used as such^37^. The dihedral angle parameters were modified to compensate for the full 1–4 scaling. The dihedral angle parameters were optimized to match the energy difference between eclipsed and staggered conformations (calculated at the HF/6-31G* level). The force field parameters for the solvents used in this study are given in Supplementary Table 1. The new parameters were validated by the liquid properties obtained from MD simulations.

Ph_2_C was also parameterized for preliminary classical MD simulations. The initial parameters were obtained using the SwissParam webserver (www.swissparam.ch/)^40^ and the partial atomic charges were calculated by fitting the restrained electrostatic potential (RESP) charges calculated at the HF/6-31G* level. The RESP charge derive (RED) webserver (http://q4md-forcefieldtools.org/RED/) was used for this purpose^41^,^42^,^43^.

### Classical MD simulations

^1^Ph_2_C was calculated under four solvent conditions, (I) MeCN, (II) MeOH, (III) MeCN/MeOH (80:20% v/v mixture) and (IV) MeCN/MeOH (99:1% v/v mixture). Set-ups I, II and III were simulated for 5 ns in a cubic periodic box at 300 K and 1 atm pressure, using 2 fs as timestep. Set-up IV was simulated for 20 ns under the same conditions. The cutoff for short-range non-bonded interactions was 10 Å. The non-bonded potential was smoothly shifted to zero at 12 Å. Particle mesh Ewald summation was used to account for long-range electrostatics. Ph_2_C was kept rigid. All the classical MD simulations were carried out using the nanoscale molecular dynamics (NAMD) program^44^.

### QM/MM MD simulations

Snapshots of Ph_2_C in a droplet of solvent (of 30 Å radius from the carbene center) were taken from the classical MD trajectories. Then, Ph_2_C in the solvent sphere was subjected to 10 ps QM/MM MD simulation, at 300 K with positional restraints on the carbene center. The QM region was formed by the Ph_2_C molecule and treated at the B3LYP-D3/def2-SVP level of theory^45^,^46^,^47^,^48^ while the solvents were treated at the MM level (see reactivity studies section for larger QM regions). The ChemShell package was used for all the QM/MM calculations, with Turbomole (v6.6) for the QM region^49^ and DL-POLY code^50^ for the MM part. The interactions between QM and MM subsystems were calculated using an electrostatic embedding scheme, in which the MM point charges polarize the QM charge density^51^.

### QM/MM optimizations

Ten snapshots taken from the last 2 ps of the QM/MM MD simulations were used for QM/MM optimizations. During the QM/MM optimization, Ph_2_C was treated at the B3LYP-D3/def2-TZVPP level^52^. QM/MM MD simulations and optimizations were performed separately for singlet and triplet states.

### Synthesis of precursor

Benzophenone (Sigma Aldrich, 99%) and p-Toluenesulfonyl hydrazide (ABCR, 98%) were used without further purification. Benzophenone tosylhydrazone was prepared according to a literature procedure^53^ by refluxing for 40 h a mixture of 1 eq. of benzophenone and 2 eq. of p-toluenesulfonyl hydrazide in absolute ethanol. The crude product was purified by recrystallization in ethanol. By treating the tosylhydrazone with 1.1 eq. of NaH (60% dispersion in mineral oil) in dry CH_2_Cl_2_ the corresponding sodium salt is formed. Sublimation of the salt on a cold substrate at 45 °C under reduced pressure yields the characteristic dark purple diphenyldiazomethane. Infrared (Ar, 3 K): 3,070 (m), 2,046 (vs), 1,598 (s), 1,582 (m), 1,502 (s), 1,497 (s), 1,457 (m), 1,447 (m), 1,320 (m), 1,268 (m), 1,262 (m), 1,034 (m), 936 (m), 756 (s), 750 (s), 697 (s), 692 (s), 651 (s), 482 (m) cm^−1^. H NMR (200 MHz, DMSO) *δ*=7.44 (m, 4H), 7.27 (m, 6H).

### Sample preparation

Even small fluctuations in the sample concentration would lead to changes in the absolute absorption-change signals and, therefore, affect the comparison of quantitative values such as the amount of Ph_2_CH^+^ produced in different solvent environments. Hence, we use a MeOH and a MeCN parent solution, respectively, each containing 294 mg Ph_2_CN_2_ in 13 ml solvent. Despite the high concentration in the latter solutions, no problems regarding solubility have been observed. For each 20 ml sample solution, 1 ml of the corresponding parent solution was extracted using a volumetric pipette. Which parent solution is taken is determined by the desired solvent ratio. In our case, concentrations between 0 to 90% MeCN were prepared using the MeOH parent solution, whereas the MeCN parent solution was the starting point for solutions containing 95 to 100% MeCN. To finally obtain the sample solutions under investigation, missing solvent was separately extracted using volumetric pipettes and added to the 1 ml of parent solution.

Solvents (Merck Millipore, Uvasol) were used as received. All samples examined were prepared for equal molarity although the molar masses of MeOH and MeCN significantly differ. Albeit both solvents have approximately the same density, the ratio of the actual number of solvent molecules of each sort differs from the associated volume ratio. Proper sample exchange was ensured by cleaning the tubing system after recording each data set with a pure solvent mixture, which corresponded to the subsequently examined sample mixture.

### TA measurements

The TA of diphenylcarbene (Ph_2_C) generated via ultraviolet photolysis of the diazo-compound precursor diphenyldiazomethane (Ph_2_CN_2_), is reported in solvent mixtures of MeOH and MeCN. All radiation employed originates from a regenerative Ti:Sapphire amplifier system (Solstice; Spectra Physics: 1 kHz, 800 nm, 100 fs). The latter is used to pump a non-collinear optical parametric amplifier (TOPAS-White; Light Conversion, Ltd) in order to generate 285 nm pump pulses. These pulses are subsequently compressed using an acousto-optic programmable dispersive filter (Dazzler; Fastlite)^54^ to a pulse duration of 40 fs at the sample position, as determined by difference-frequency cross-correlation frequency-resolved optical gating (XFROG) measurements^55^,^56^. Another fraction of the 800 nm fundamental beam is focused into a linearly moving CaF_2_ plate to generate supercontinuum probe pulses with spectral components down to ≈300 nm. The beams, focused to diameters of roughly 50 μm (pump) and 40 μm (probe), respectively, are spatially overlapped at the sample position, whereas the polarization directions are held at the magic angle configuration (54.7°) (ref. 57). For excitation, 130 nJ pump pulses are applied to stay in the linear excitation regime (Supplementary Fig. 18 and Supplementary Note 10), whereas the energy of the probe pulses is substantially lower. A spectrally resolved shot-to-shot detection of changes in optical density between the pumped and unpumped probe volume is enabled by using a visible spectrograph (Acton SP2500i; Princeton Instruments) combined with a two-dimensional CCD (charge-coupled device) camera (Pixis 2 K; Princeton Instruments) with an acquisition rate of 1 kHz and mechanically chopping the pump pulses at 500 Hz. To exchange the probed sample volume between subsequent pump-probe pairs, a 20 ml sample volume is perpetually pumped through a Suprasil flow cell of 200 μm path length, whereby 6 mM solutions of the precursor are employed, resulting in an optical density of about 2 at 285 nm along the flow cell. Measurement times are kept sufficiently short to prevent major sample degradation due to continuous photolysis of the precursor, as verified by measurements of the linear absorption spectrum before and after the time-resolved experiments. In the measurement routine, the pump-probe delay is scanned up to 4 ns using linear step sizes within the first few picoseconds followed by exponential step sizes, whereas the average of 1,000 consecutive TA spectra is recorded at each time delay. No mechanical changes in the set-up-alignment were made when recording TA data for a total of 21 sample solutions. All data shown results from averaging over three subsequently recorded data sets in order to minimize long-term fluctuations. The data has been corrected for the chirp of the supercontinuum probe^57^.

### Data availability

Data supporting the findings of this study are available within the article and Supplementary Information, as well as from the corresponding authors upon reasonable request.

## Additional information

**How to cite this article:** Knorr, J. *et al*. Competitive solvent–molecule interactions govern primary processes of diphenylcarbene in solvent mixtures. *Nat. Commun.* **7,** 12968 doi: 10.1038/ncomms12968 (2016).

## Supplementary Material

Supplementary Information Supplementary Figures 1-18, Supplementary Tables 1-10, Supplementary Notes 1-10 and Supplementary References

Supplementary Movie 1 Full set of transient absorption measurements (285 nm pump pulses) on Ph2CN2 dissolved in 21 different MeOH/MeCN solvent mixtures: (left) data shown using a linear time axis covering the first 135 ps pump-probe delay; (right) data shown using a lin-log time axis in order to include dynamics up to 4 ns pump-probe delay. Numbers depict the volume fraction of solvents in the respective mixture.
