# Supplementary material for: Competitive solvent-molecule interactions govern primary processes of diphenylcarbene in solvent mixtures
Source: Nat Commun. 2016 Oct 6;7:12968. doi: 10.1038/ncomms12968 (PMC5059701; doi:10.1038/ncomms12968)
Supplement: Supplementary Information — Supplementary Figures 1-18, Supplementary Tables 1-10, Supplementary Notes 1-10 and Supplementary References [file ncomms12968-s1.pdf]

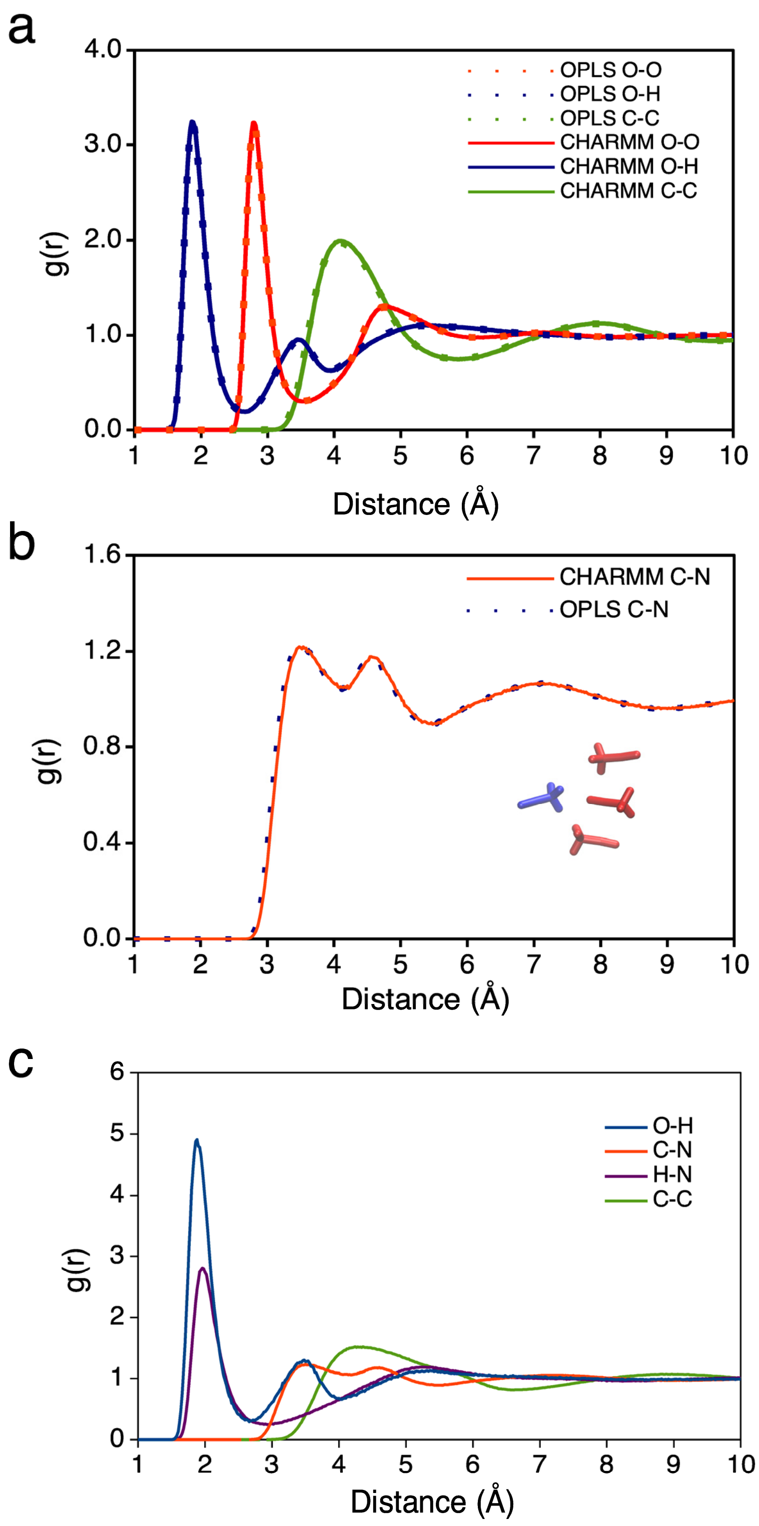

Supplementary Figure 1: **Calculated radial distribution functions (RDF).** (**a**) RDF of selected pairs of atoms in methanol. The solid line indicates the CHARMM simulation and the dotted line indicates the OPLS simulation. (**b**) RDF of the C--N pair in MeCN: CHARMM simulation (solid line) and OPLS simulation (dotted line). The two orientations of MeCN observed in the MD simulation are shown in the inset. The antiparallel orientation is shown by red sticks and the parallel (head-to-tail) orientation is shown by blue and red sticks in the center. (**c**) RDF of methanol-methanol, acetonitrile-acetonitrile, and acetonitrile-methanol pairs in the 80:20% v/v mixture.

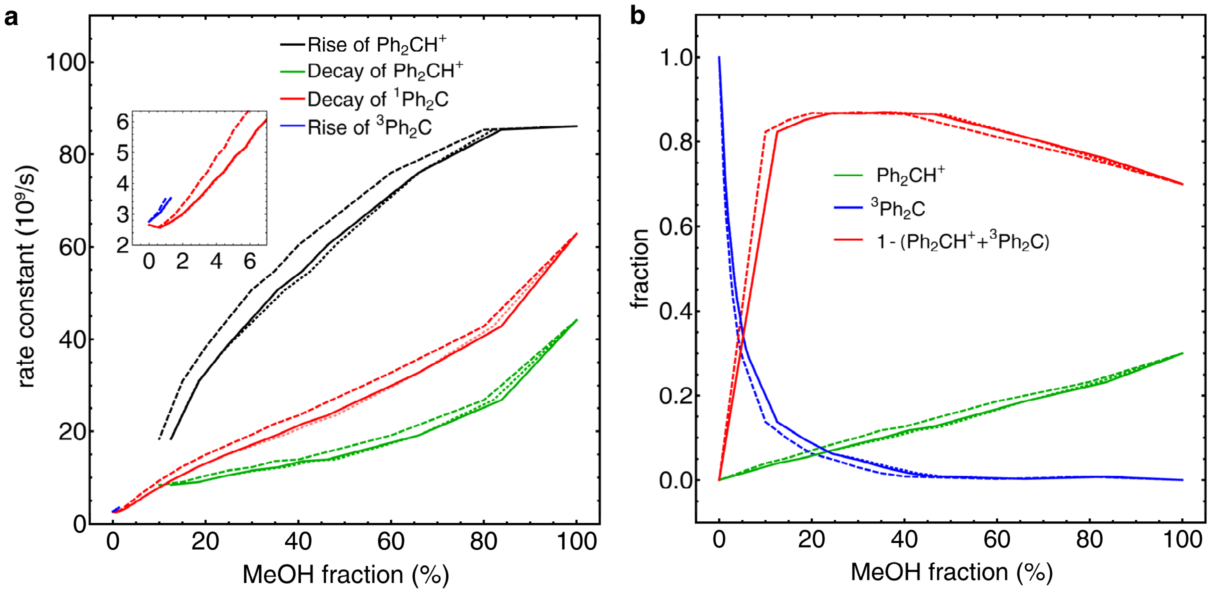

Supplementary Figure 2: **Considerations on preferential solvation.** Rate constants [(**a**), refer to Figure 4a of the main manuscript] and signal fractions [(**b**), refer to Figure 5a of the main manuscript] versus the bulk MeOH mole fraction (solid lines), the local MeOH mole fraction (dotted lines), and the MeOH volume fraction (dashed lines) in the solvent environment.

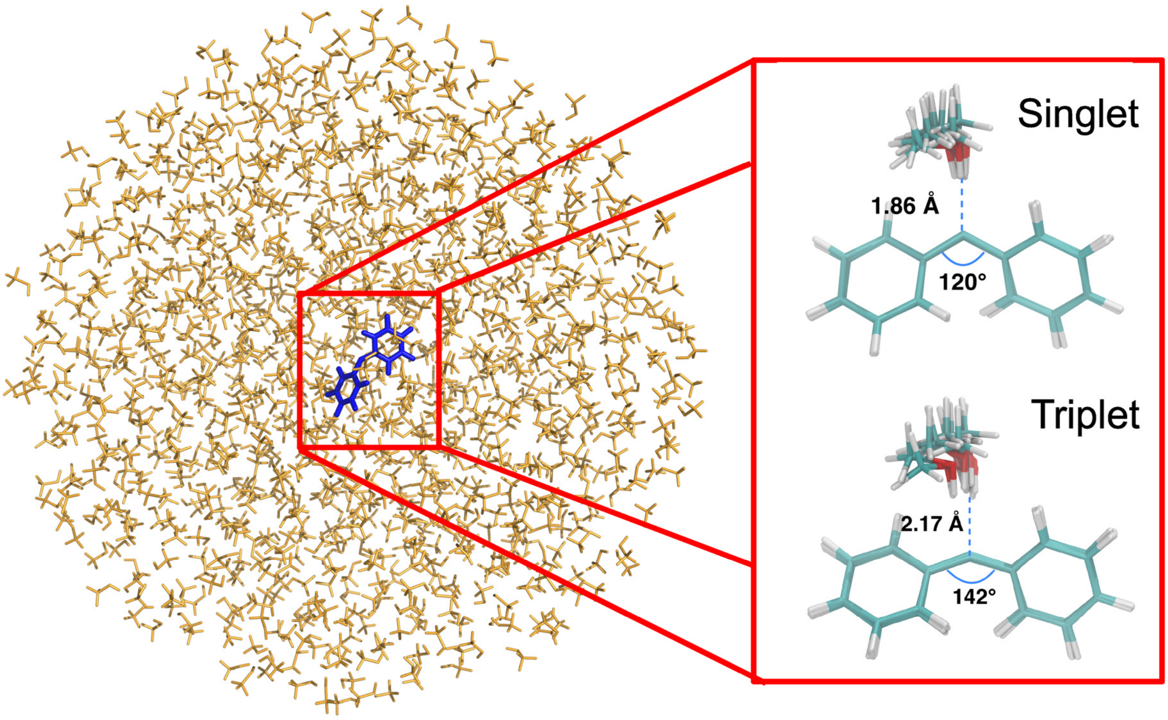

Supplementary Figure 3: **Hydrogen bonding between Ph_2C and methanol.** The carbene molecule is placed at the center of a solvent droplet in QM/MM calculations.

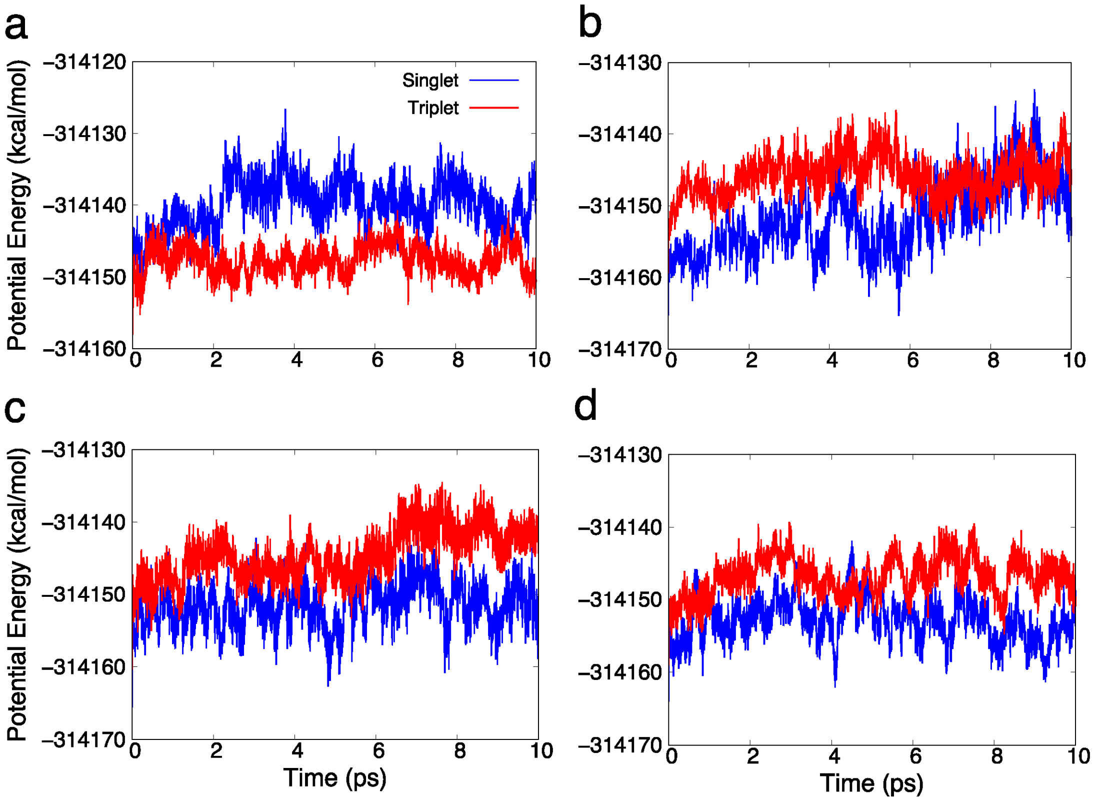

Supplementary Figure 4: **Distribution of singlet and triplet energies during QM/MM MD simulations**. Calculations correspond to Ph_2C in (**a**) acetonitrile, (**b**) methanol, (**c**) 80:20% mixture and (**d**) 99:1% mixture.

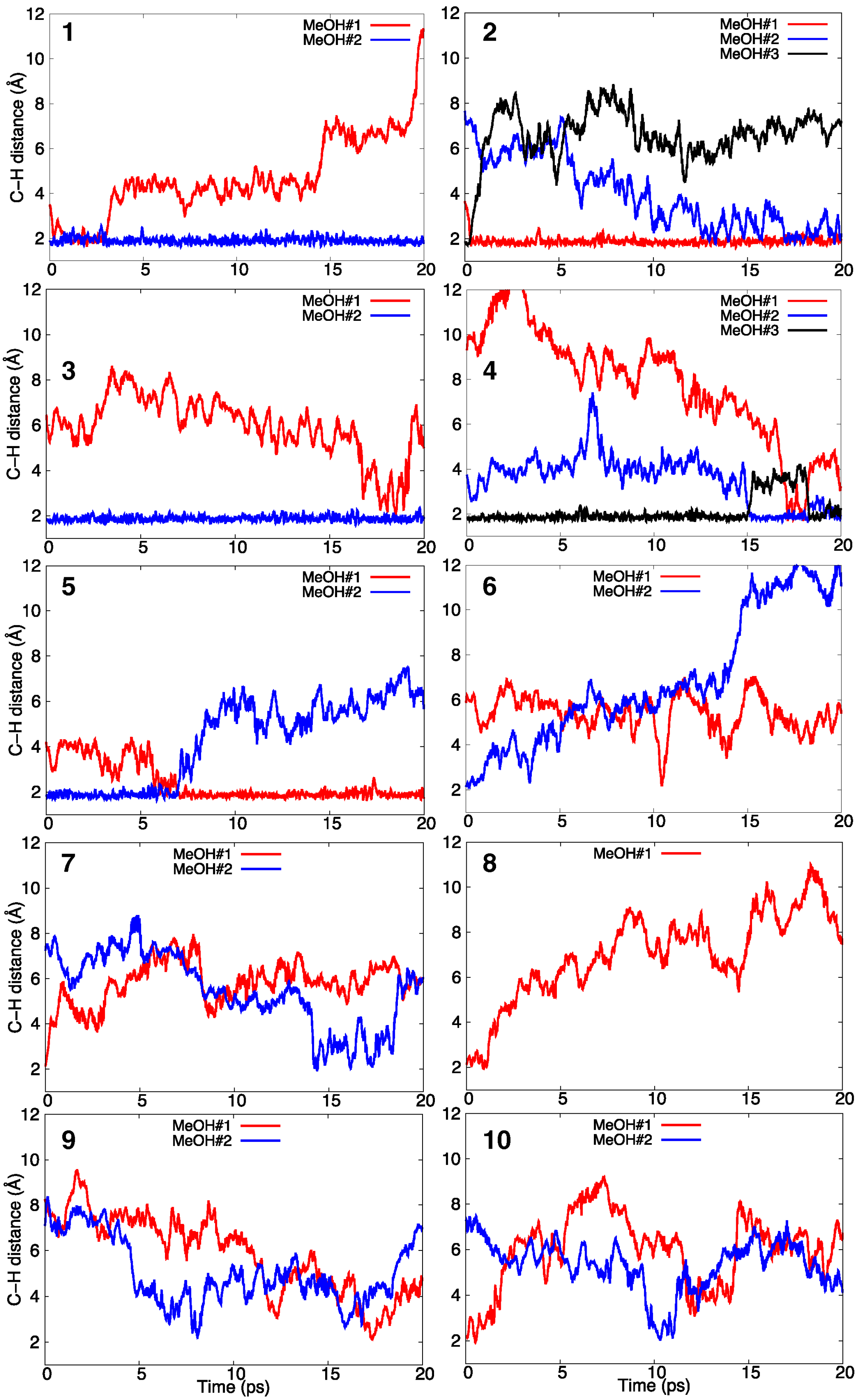

Supplementary Figure 5: **Lifetime of Ph_2C···HOMe complexes.** Results from simulations for the $^1Ph_2C$···HOMe complex (1-5) and for the $^3Ph_2C$···HOMe complex (6-10).

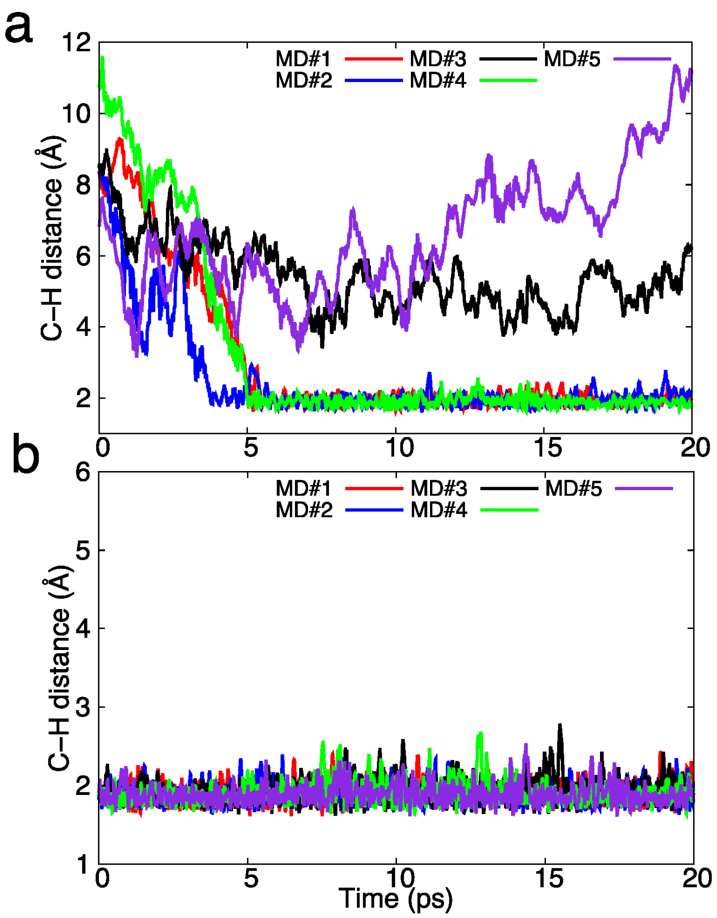

Supplementary Figure 6: **$^{1}Ph_2C^{\cdots}HOMe$ complex in the 80:20% mixture.** (**a**) Simulations performed with unbounded $^{1}Ph_2C$ and (**b**) simulations starting with the preformed $^{1}Ph_2C^{\cdots}HOMe$ complex.

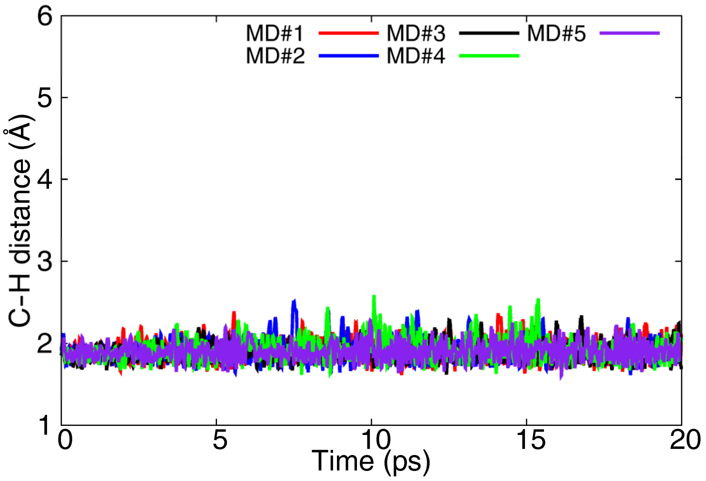

Supplementary Figure 7: **Simulations of the preformed $^{1}Ph_2C^{\cdots}HOMe$ complex in the 99:1 mixture.** In the 99:1% mixture, no $^{1}Ph_2C^{\cdots}HOMe$ complex was found in the timescale of the simulations if no MeOH molecule was within 6 Å radius of the carbene center. However, once formed, the $^{1}Ph_2C$ -MeOH complex is conserved.

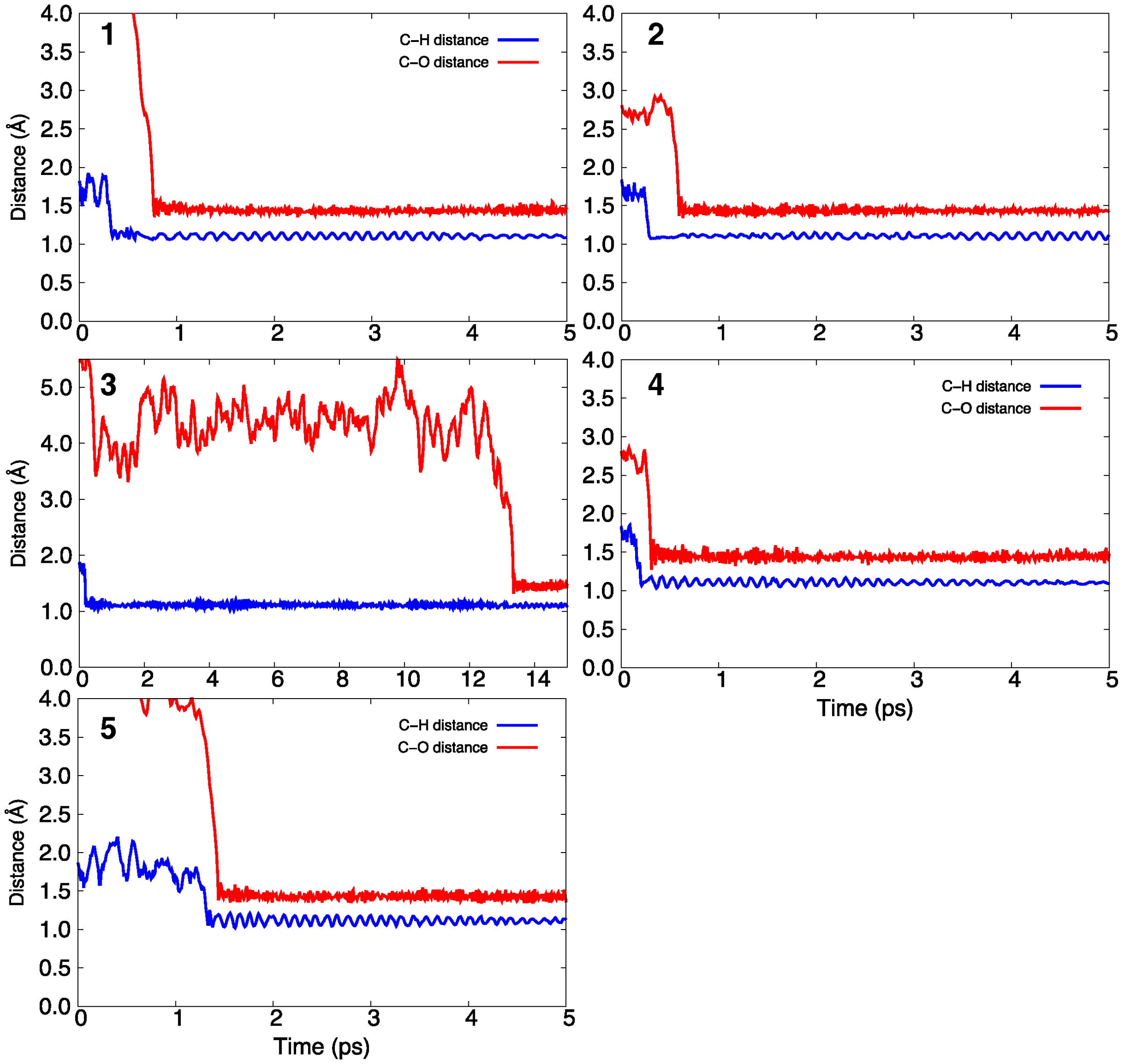

Supplementary Figure 8: **O–H insertion reaction of $^{1}Ph_2C$**. The reaction is observed in the QM/MM MD simulations through the change in C–H and C–O distances.

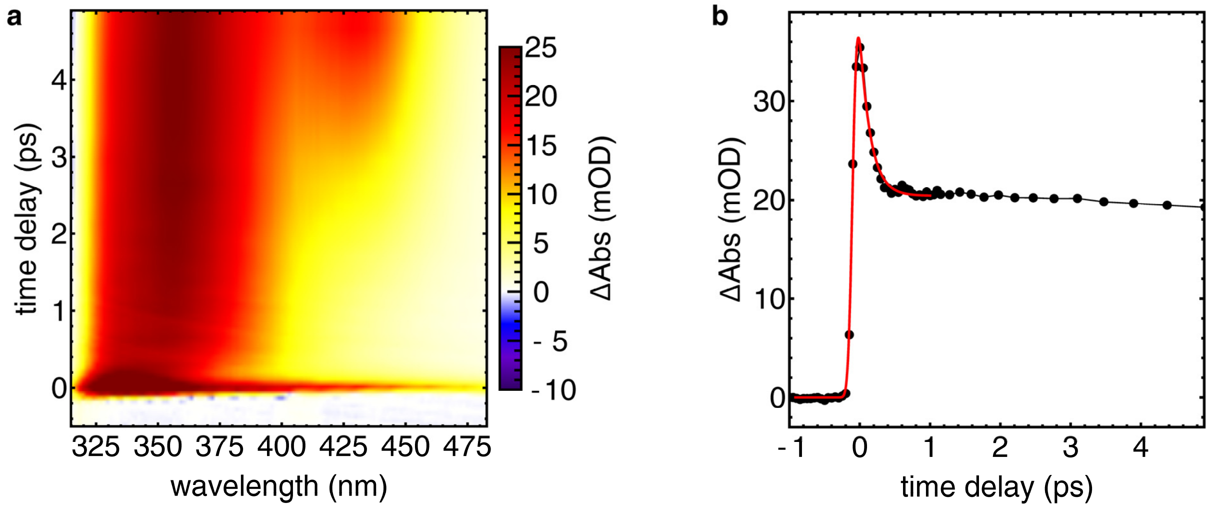

Supplementary Figure 9: **Transient absorption during the first 5 ps after UV-excitation of Ph_2CN_2 in MeOH.** (**a**) Full data set. (**b**) The transient absorption at a probe wavelength of 335 nm.

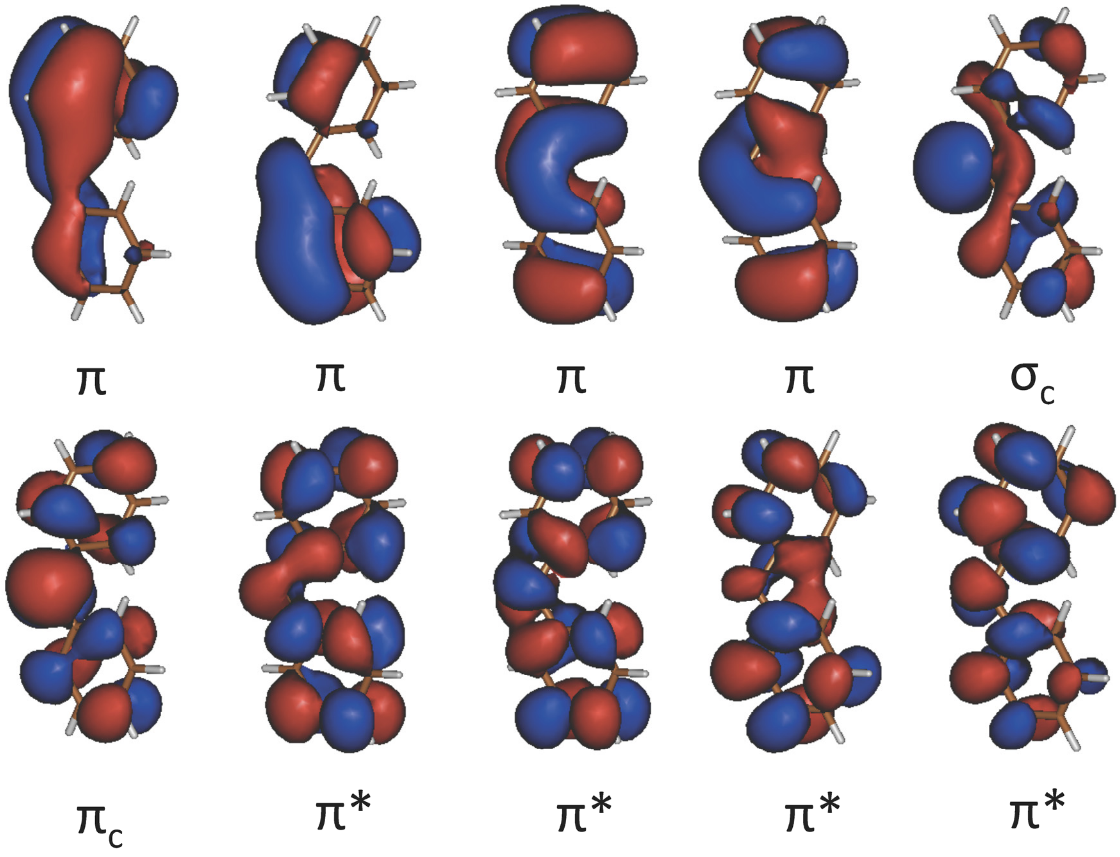

Supplementary Figure 10: **Active space used for NEVPT2/CASSCF(10,10) calculations**. NEVPT2/CASSCF(10,10) single point calculations on the B3LYP-D3/Def2-TZVPP geometries were consistent with an experimental electronic absorption spectrum of uncomplexed $^1Ph_2C$ being similar to those of both $^1Ph_2C\cdots HOH$ and $^1Ph_2C\cdots HOMe$ (see Supplementary Note 7).

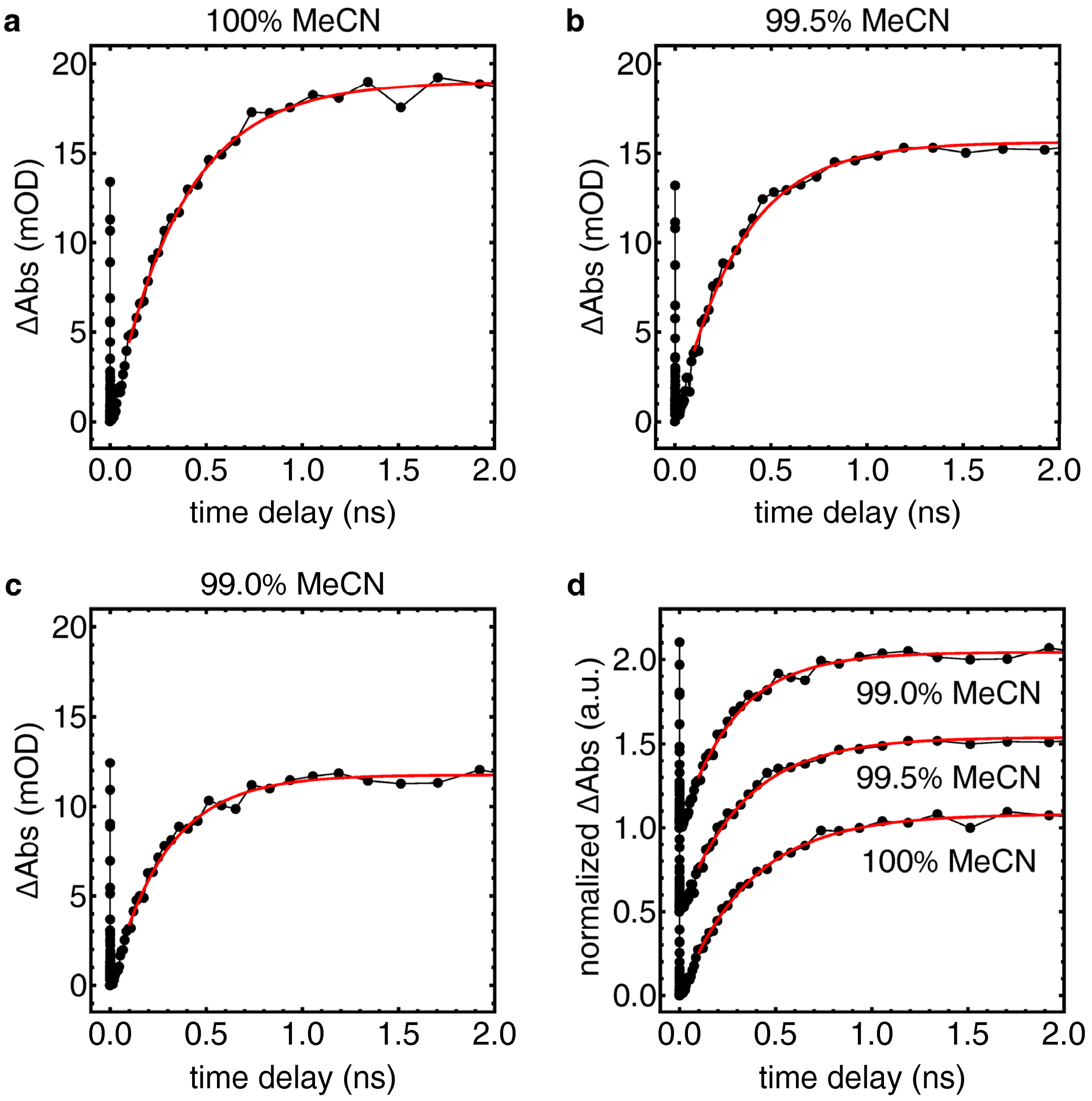

Supplementary Figure 11: **Transient absorption of Ph_2CN_2 under 285 nm excitation**. Experiments are performed for (**a**) pure MeCN and with small admixtures of (**b**) 0.5% and (**c**) 1.0% MeOH at 315 nm. For better comparability, a normalized representation is given in (**d**). The upcoming absorption of $^3Ph_2C$ is fitted using a monoexponential model function. The corresponding time constants can be found in Supplementary Table 7.

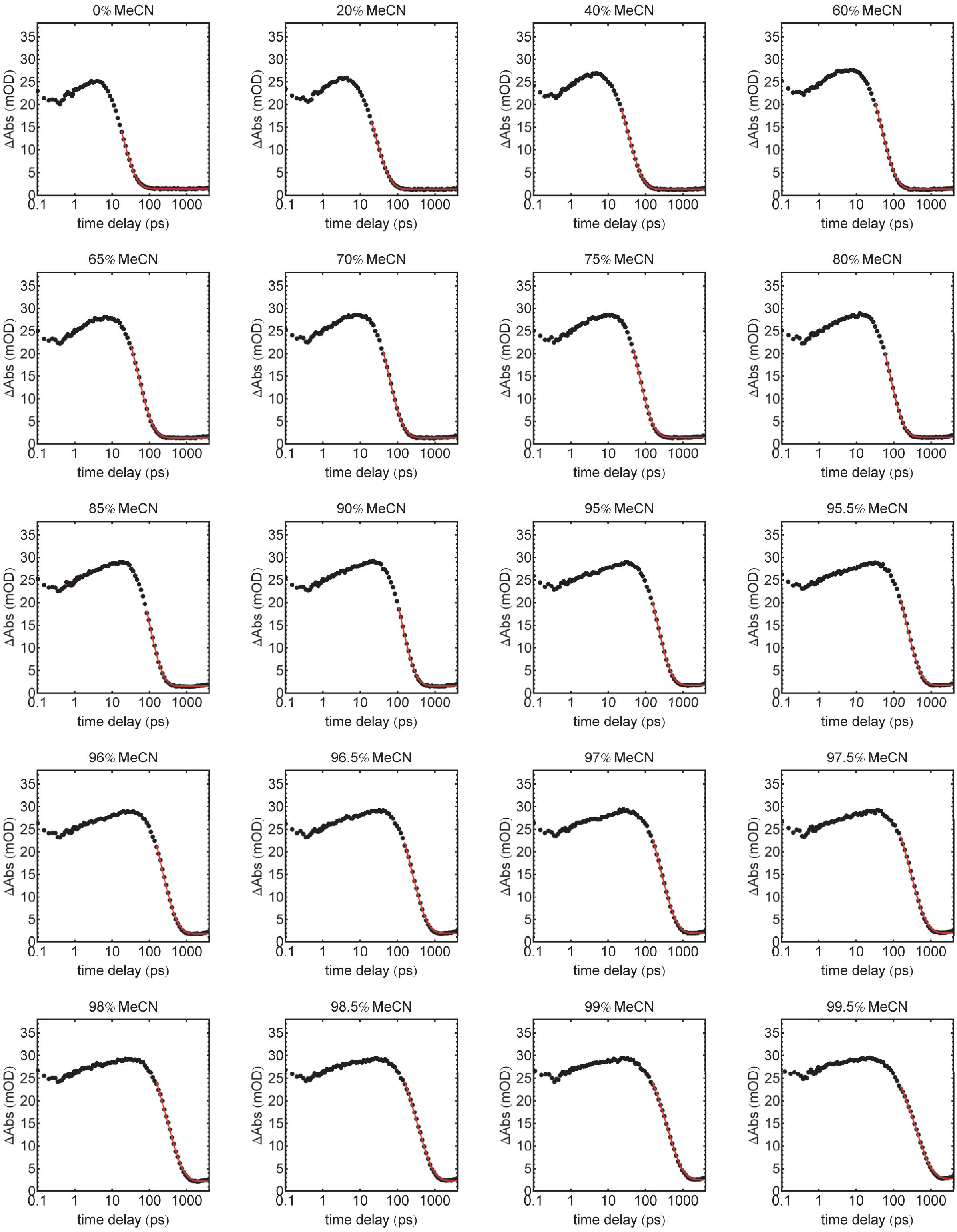

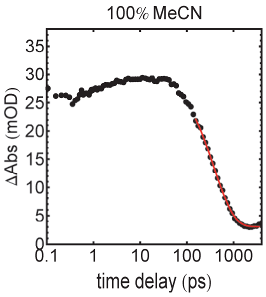

Supplementary Figure 12: **Transient absorption of Ph_2CN_2 under 285 nm excitation probed at 355 nm**. The different panels correspond to measurements with different solvent mixtures of MeOH and MeCN, as indicated.

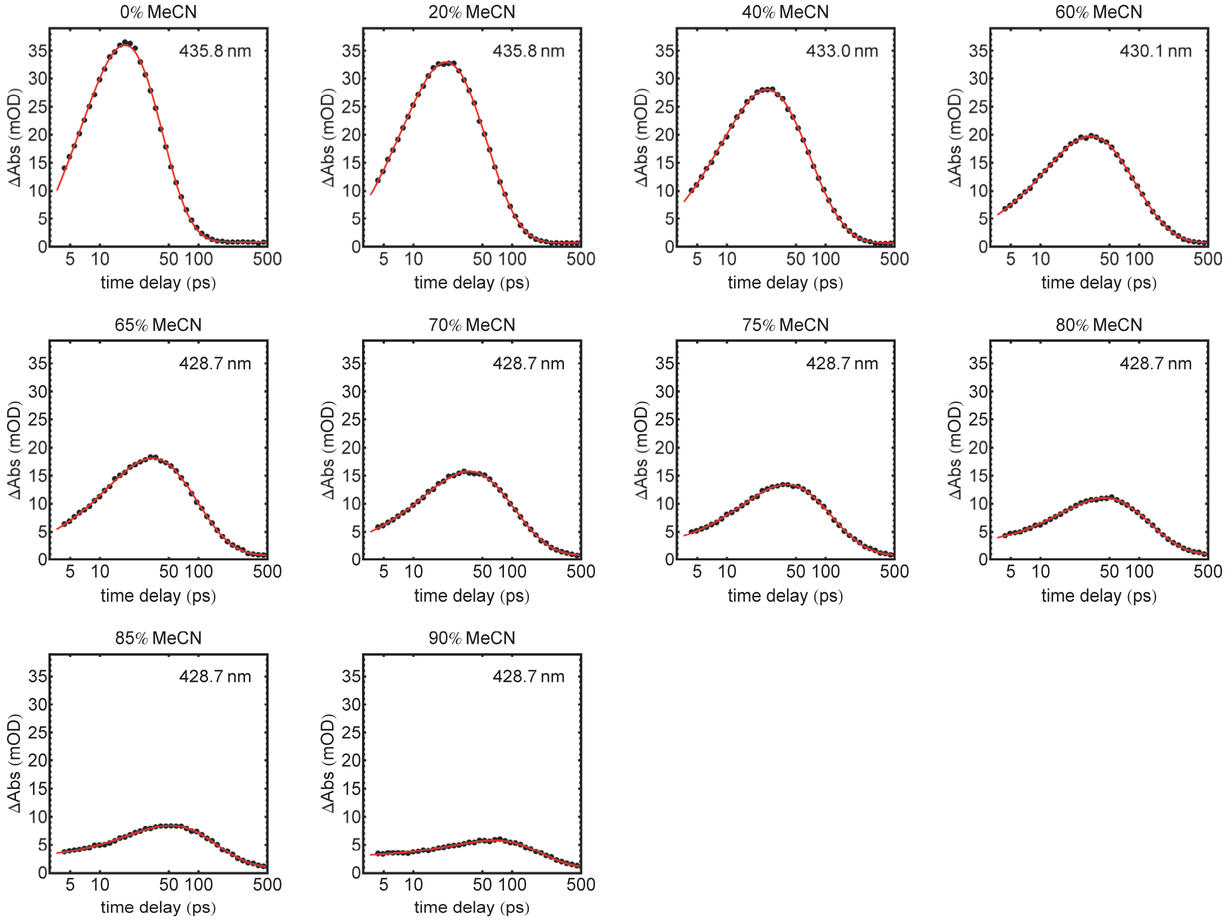

Supplementary Figure 13: **Transient absorption of Ph_2CN_2 under 285 nm excitation probed at the Ph_2CH^+ absorption.** The different panels correspond to measurements with different solvent mixtures of MeOH and MeCN at the respective center wavelengths of Ph_2CH^+, as indicated.

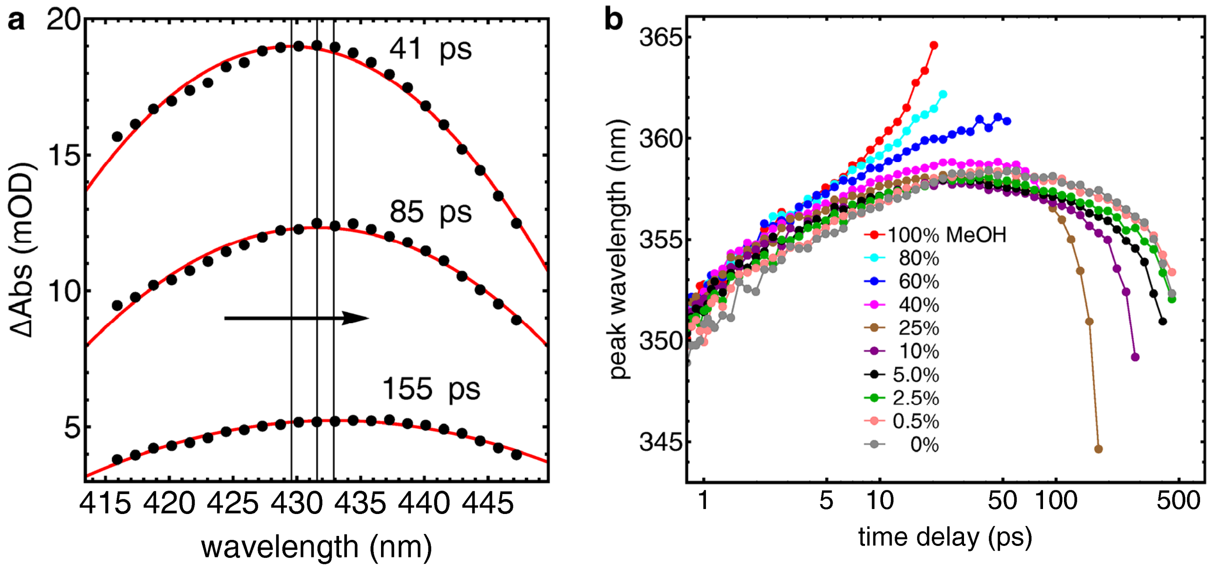

Supplementary Figure 14: **Peak shift analysis.** (**a**) Ph_2CH^+ absorption band after 285 nm excitation of Ph_2CN_2 in a 60:40 solvent mixture of MeCN:MeOH for different time delays. Displaced maxima of the fitted parabola curves (red) illustrate a spectral red-shift for increasing time delays. (**b**) Time-dependent peak position of the $^1Ph_2C$ absorption band. For all solvent environments, an initial red-shift is observed, which is followed by a much slower blue-shift for those mixtures where the signal does not vanish on the time scale of Ph_2CH^+ formation. Note the logarithmic abscissa.

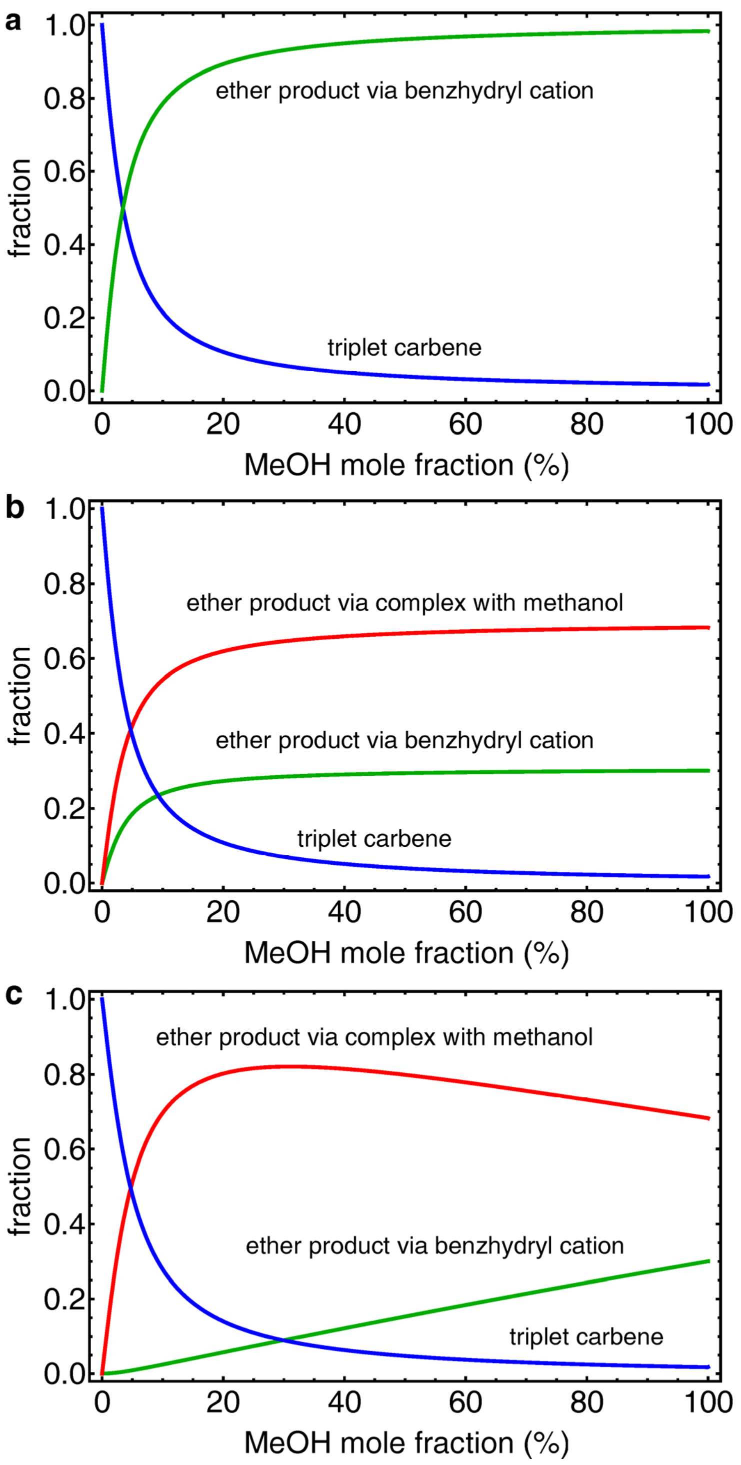

Supplementary Figure 15: **Rate-model analysis of molecule fractions following certain reaction paths.** (**a**) Percentage of molecules taking the $^{1}Ph_2C \rightarrow {}^{3}Ph_2C$ or the $^{1}Ph_2C \rightarrow Ph_2CH^{+} \rightarrow$ ether reaction path, according to model 1 in Supplementary Note 9. (**b**) Percentage of molecules taking the $^{1}Ph_2C \rightarrow {}^{3}Ph_2C$, the $^{1}Ph_2C \rightarrow Ph_2CH^{+} \rightarrow$ ether, or the $^{1}Ph_2C \rightarrow {}^{1}Ph_2C^{\cdot\cdot}HOMe \rightarrow$ ether reaction path, according to model 2 with rate constants linearly depending on [M] for the first step of the latter two reaction paths.(**c**) Percentage of molecules taking the $^{1}Ph_2C \rightarrow {}^{3}Ph_2C$, the $^{1}Ph_2C \rightarrow Ph_2CH^{+} \rightarrow$ ether, or the $^{1}Ph_2C \rightarrow {}^{1}Ph_2C^{\cdot\cdot}HOMe \rightarrow$ ether reaction path. The dependence on the MeOH concentration for the initial rate of each reaction path is zero, quadratic, and linear, respectively.

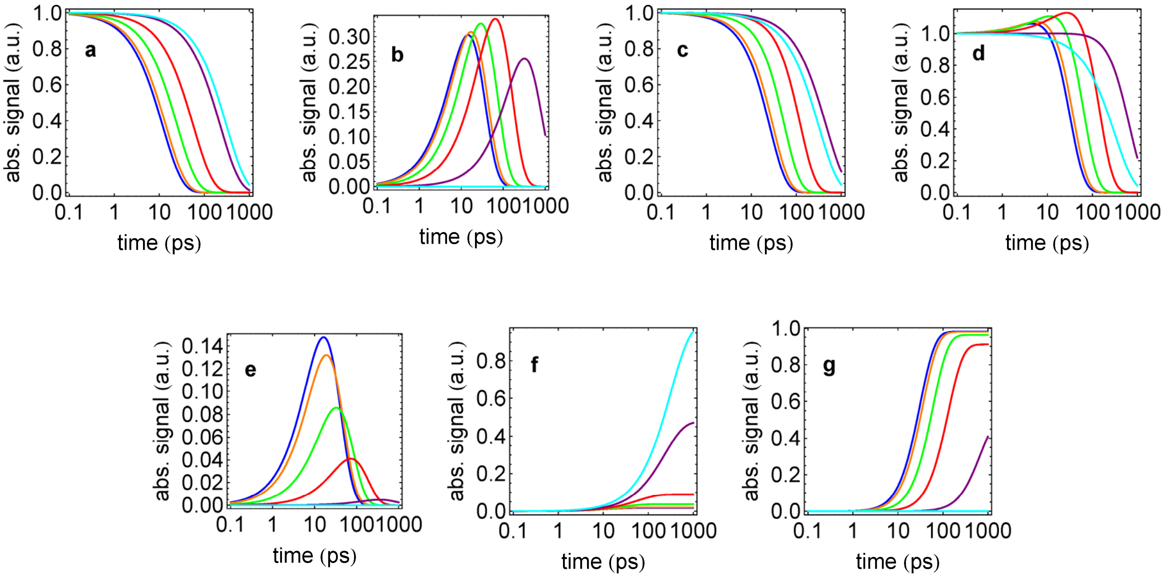

Supplementary Figure 16: Modeled transient absorption signals. The data are calculated for a MeOH mole fraction of 0.0 (blue), 0.1 (orange), 0.4 (green), 0.7 (red), 0.95 (purple), and 1.0 (cyan). The curves correspond to the signals of (**a**) $^1Ph_2C$, (**b**) $^1Ph_2C\cdots HOMe$, (**c**) $^1Ph_2C$ + $^1Ph_2C\cdots HOMe$, and (**d**) $^1Ph_2C$ + $^1Ph_2C\cdots HOMe$ with the latter contribution multiplied by a factor of 2 to mimic a higher absorption coefficient. Lower panels: (**e**) Ph_2CH^+, (**f**) $^3Ph_2C$, and (**g**) ether product.

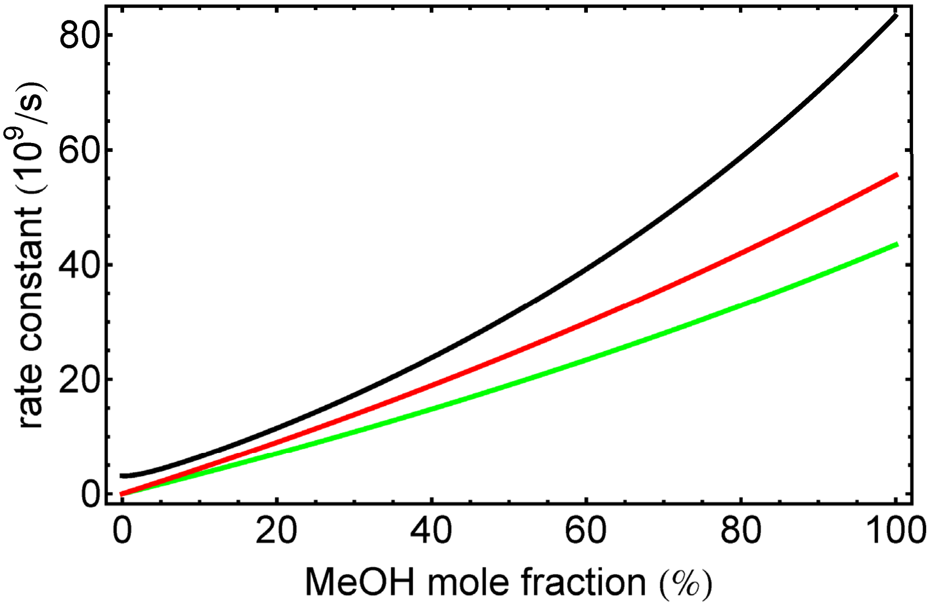

Supplementary Figure 17: **Modeled rate constants as a function of solvent mixing ratio**. The data corresponds to the rise of the Ph_2CH^+ signal (black), the decay of Ph_2CH^+ (green), and the decay of $^1Ph_2C\cdots HOMe$ (red).

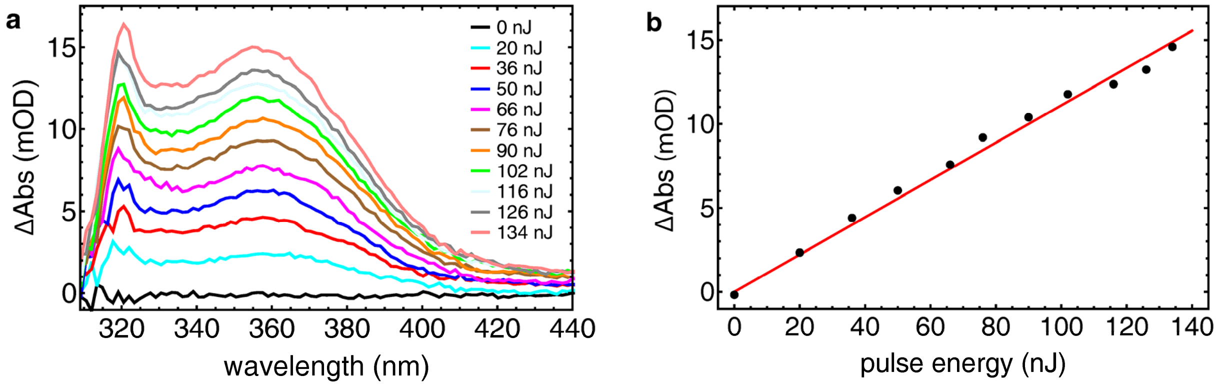

Supplementary Figure 18: **Dependence of transient absorption signal on pump-laser intensity variation.** (**a**) Transient absorption spectra of Ph_2CN_2 in MeCN at 360 ps pump-probe delay for various 285 nm pump-pulse energies and (**b**) linear fit of the corresponding absorption change signals at 360 nm versus the employed pulse energy.

Supplementary Table 1: Force field parameters for MeOH and MeCN.

**MeOH**			
**Bonds**	**b_0 (Å)**	**K_b (kcal mol^{-1} Å^{-2})**	
C-H<br>C-O<br>O-H	1.090<br>1.410<br>0.945	340.000<br>320.000<br>553.000	
**Angles**	**θ_0 (degrees)**	**K_θ (kcal mol^{-1} rad^{-2})**	
H-C-H<br>H-C-O<br>C-O-H	107.8<br>109.5<br>108.5	33.000<br>35.000<br>55.000	
**Dihedrals**	**δ (degrees)**	**K_φ (kcal mol^{-1})**	**n**
H-C-O-H	0.0	0.222	3
**Non-bonded**	**ε (kcal mol^{-})**	**$r_{min}/2$ (Å)**	**Partial charge**
H<br>C<br>O<br>H (hydroxyl)	-0.030<br>-0.066<br>-0.170<br>-0.000	1.403<br>1.964<br>1.751<br>0.000	0.040<br>0.145<br>-0.683<br>0.418

**MeCN**			
**Bonds**	**b_0 (Å)**	**K_b (kcal mol^{-1} Å^{-2})**	
C-H<br>C-C<br>C-N	1.090<br>1.458<br>1.157	340.000<br>385.000<br>650.000	
**Angles**	**θ_0 (degrees)**	**K_θ (kcal mol^{-1} rad^{-2})**	
H-C-H<br>H-C-C<br>C-C-N	107.8<br>108.5<br>180.0	33.000<br>35.000<br>150.000	
**Non-bonded**	**ε (kcal mol^{-1})**	**$r_{min}/2$ (Å)**	**Partial charge**
H<br>C<br>C (nitrile)<br>N	-0.015<br>-0.066<br>-0.066<br>-0.170	1.403<br>1.852<br>1.852<br>1.796	0.060<br>-0.080<br>0.460<br>-0.560

Supplementary Table 2: ΔE_{ST} of Ph_2C in explicit acetonitrile (QM/MM optimizations).

**Snapshot**	**Singlet energy (kcal mol^{-1})**	**Triplet energy (kcal mol^{-1})**
1	-314499.0	-314499.5
2	-314498.1	-314499.8
3	-314498.4	-314500.0
4	-314498.6	-314501.4
5	-314498.8	-314500.2
6	-314497.8	-314500.5
7	-314499.2	-314501.7
8	-314496.2	-314501.4
9	-314497.5	-314499.6
10	-314501.3	-314500.5
Mean	-314498.5	-314500.5
Std. dev.	1.3	0.8
ΔE_{ST} (calc.)	2.0 kcal mol^{-1}	
ΔE_{ST} (expt.)[a]	2.63 kcal mol^{-1}	

[a]Ref. 1

Supplementary Table 3: ΔE_{ST} of Ph_2C in explicit methanol (QM/MM optimizations).

**Snapshot**	**Singlet energy (kcal mol^{-1})**	**Triplet energy (kcal mol^{-1})**
1	-314508.9	-314499.9
2	-314508.4	-314501.0
3	-314507.9	-314501.5
4	-314506.8	-314501.7
5	-314508.6	-314502.3
6	-314508.2	-314501.2
7	-314505.4	-314501.1
8	-314510.2	-314499.4
9	-314510.5	-314499.8
10	-314508.7	-314499.2
Mean	-314508.4	-314500.7
Std. dev.	1.5	1.1
ΔE_{ST} (calc.)	-7.7 kcal mol^{-1}	

Supplementary Table 4: ΔE_{ST} in acetonitrile-methanol mixtures.

	80:20 % mixture (kcal mol^{-1})		99:1 % mixture (kcal mol^{-1})	
**Snapshot**	**Singlet**	**Triplet**	**Singlet**	**Triplet**
1	-314509.2	--	-314507.7	-314498.2
2	-314510.0	-314499.3	-314512.4	-314498.8
3	-314508.9	-314501.0	-314512.4	-314499.7
4	-314505.1	-314498.0	-314508.5	--
5	-314508.7	-314499.3	-314508.3	-314500.6
6	-314509.7	--	-314510.6	-314498.9
7	-314510.5	-314500.9	-314510.6	-314500.5
8	-314509.6	-314499.6	-314508.3	-314501.3
9	-314509.1	-314498.2	-314508.6	-314501.3
10	-314511.5	-314499.1	-314507.8	-314499.3
Mean	-314509.2	-314499.4	-314509.5	-314599.8
Std. dev.	1.7	1.1	1.8	1.1
ΔE_{ST}	-9.8 kcal mol^{-1}		-9.7 kcal mol^{-1}	

Supplementary Table 5: UV-visible transitions for $^1Ph_2C$ and hydrogen bonded complexes of $^1Ph_2C$ calculated at the NEVPT2/CASSCF(10,10) level of theory.

**System**	**NEVPT2/CASSCF UV-visible transitions (nm)**	**Oscillator strength**
$^1Ph_2C$	423	0.017
	349	0.058
	326	0.006
$^1Ph_2C\cdots HOH$	390	0.005
	329	0.095
	296	0.024
$^1Ph_2C\cdots HOMe$	390	0.004
	328	0.092
	302	0.012
$^1Ph_2C\cdots HOMe$ (QM/MM)	389	0.005
	328	0.096
	300	0.017

Supplementary Table 6: Fit of the Ph_2CH^+ peak position.

MeCN (%)	A_1 (nm)	A_2 (nm)	τ (ps)	t_0 (ps)
0	-2.6	0	23.5	11.2
20	-3.0	0.37	36.1	11.2
40	-3.9	0.29	46.5	12.6
60	-5.6	0.26	69.7	18.0
65	-6.1	1.15	85.7	20.2
70	-7.6	2.83	130.5	29.0
75	-6.6	1.53	110.3	32.6

Fit of the Ph_2CH^+ peak position in MeOH/MeCN solvent mixtures using the model function $\lambda(t) = A_1 \exp(-(t - t_0)/\tau) + A_2$.

Supplementary Table 7: Time constants obtained by fitting the rise of $^3Ph_2C$ in solvent mixtures of MeOH and MeCN (spectroscopic grade).

**MeCN (%)**	100	99.5	99.0
**τ (ps)**	361.8	328.7	283.5

Supplementary Table 8: Parameters of fitting the decay of $^1Ph_2C$.

**MeCN (%)**	0	20	40	60	65	70	75
**τ (ps)**	15.9	23.4	30.5	42.7	46.1	51.8	58.3

**MeCN (%)**	80	85	90	95	95.5	96.0	96.5
**τ (ps)**	67.0	80.6	107.1	175.4	193.5	205.3	227.6

**MeCN (%)**	97.0	97.5	98.0	98.5	99.0	99.5	100
**τ (ps)**	246.0	274.1	302.2	336.3	363.7	390.8	376.5

Time constants obtained by fitting the decay of $^1Ph_2C$ in MeOH/MeCN solvent mixtures using the model function $\Delta Abs(t) = A_1 \exp(-(t - t_0)/\tau) + A_2$.

Supplementary Table 9: Parameters of fitting the rise and decay of Ph_2CH^+.

MeCN (%)	0	20	40	60	65	70	75	80	85	90
λ (nm)	435.8	435.8	433.0	430.1	428.7	428.7	428.7	428.7	428.7	428.7
τ_1 (ps)	11.6	11.7	13.2	16.6	18.2	19.7	22.4	26.1	32.3	54.5
τ_2 (ps)	22.7	37.4	52.2	71.9	73.6	81.7	86.6	96.2	110.5	118.6

Time constants obtained by fitting the rise and decay of Ph_2CH^+ in MeOH/MeCN solvent mixtures using the model function $\Delta Abs(t) = A_1 \; \exp(-(t - t_0)/\tau_1) - A_2 \exp(-(t - t_0)/\tau_2) + A_3$.

Supplementary Table 10: Amount of molecules following the three reaction paths.

MeOH mole fraction (%)	ether product via complex with methanol	ether product via benzhydryl cation	triplet carbene
0.0	0.0	0.0	100.0
0.6	-	-	86.6
1.3	-	-	71.6
1.9	-	-	62.1
2.6	-	-	55.5
3.2	-	-	48.3
3.8	-	-	42.9
4.5	-	-	39.0
5.1	-	-	34.4
5.7	-	-	31.3
6.4	-	-	29.1
12.5	82.3	4.0	13.7
18.6	85.1	5.3	9.6
24.4	86.8	7.0	6.2
30.1	86.7	8.6	4.7
35.6	86.9	10.1	3.0
41.0	86.6	11.8	1.6
46.3	86.5	12.6	0.9
65.9	81.1	18.6	0.3
83.8	75.9	23.3	0.8
100.0	70.0	30.0	0.0

Fraction of molecules following the $^1Ph_2C\cdots HOMe$, the Ph_2CH^+, and the $^3Ph_2C$ pathway for different MeOH mole fractions, as derived from the transient absorption data. For relative amounts below 4% of the benzhydryl cation, the determination from the experimental data was less reliable, as indicated by a dash. Note the remarks in the main manuscript for the values in pure solvents.

Supplementary Note 1: Validation of solvent parameters

NPT simulations of pure MeOH in a cubic box were carried out for 10 ns at 298 K and 1 atm pressure to validate the new parameters. As a reference, the simulation was carried out under the same conditions using the OPLS force field as implemented in the GROMACS code (v4.6).[2] The physical properties of the solvents calculated using the CHARMM and OPLS force fields were compared. The density of MeOH from the CHARMM simulation (0.769 g/cm^3) is in good agreement with that from the OPLS simulation (0.774 g/cm^3) as well as with the experimental density (0.791 g/cm^3 at 298 K). The radial distribution of selected pair of atoms (O--O, O--H and C--C pairs) averaged over the simulation trajectory is shown in Supplementary Figure 1a. The calculated radial distribution functions (RDF) are very similar in both CHARMM (solid lines in Supplementary Figure 1a) and OPLS (dotted lines in Supplementary Figure 1a) calculations, indicating that the solvent structure and hydrogen bonding were not affected by the conversion of parameters.

The NPT simulations of MeCN (10 ns at 298 K and 1 atm pressure) showed a reasonable agreement between CHARMM and OPLS densities (0.745 g/cm^3 and 0.765 g/cm^3, respectively). The RDF of the C-N pair in MeCN showed two less pronounced peaks at short range (3.5 Å and ~4.5 Å), indicating the absence of a well-structured solvent arrangement (Supplementary Figure 1b). MeCN molecules interact with each other in two different orientations, i) head-to-tail type parallel (RDF peak at 3.5 Å) and ii) antiparallel (RDF peak at ~4.5 Å). These two orientations are shown in Supplementary Figure 1b (inset).

Finally, the solvent parameters were tested using a mixture of MeCN and MeOH (80:20% v/v). The RDF of the mixture showed not only methanol-methanol (O--H) and acetonitrile-acetonitrile (C--N) peaks, but also high intensity acetonitrile-methanol (N--H and C--C) peaks. This indicates a good miscibility of the two solvents (Supplementary Figure 1c). Furthermore, all the RDF curves converged to unity at long distances (>10 Å), which indicates the absence of phase separation or aggregate formation.

Supplementary Note 2: Preferential solvation in solvent mixtures

The effect of preferential solvation in binary solvent mixtures containing MeOH and MeCN is discussed in a theoretical study by Marcus.[3] It was shown that on the microscopic level, such a solvent mixture behaves more complex than just an even distribution of molecules of each sort. Instead, solvent molecules of each sort prefer to keep among themselves to a certain extent, leading to the formation of small solvent clusters resulting in differences between the bulk and the local mole fractions, respectively. In Supplementary Figure 2, we juxtapose an analysis including the preferential solvation according to the method of inverse Kirkwood-Buff integrals (IKBI) of Ref. 3 to the data from Figures 4a and 5a of the main manuscript. Furthermore, in Supplementary Figure 2 the data is presented when plotted against the volume rather than the mole fractions. As can be seen from the rather small differences, the discussion and interpretation of the experimental findings is unaffected by including these effects or presenting the data as a function of volume fraction.

Supplementary Note 3: Singlet-triplet energy gap (ΔE_{ST})

Pure acetonitrile

As mentioned above, $^1Ph_2C$ was solvated in a cubic box of MeCN and subjected to an MD simulation at 300 K to equilibrate the solvent. A snapshot of $^1Ph_2C$ in a droplet of solvent within 30 Å radius of the carbene center was used for 10 ps QM/MM MD simulations. Ten snapshots were then optimized with $^1Ph_2C$ ($^3Ph_2C$) as QM region (B3LYP-D3/def2-TZVPP level of theory) and acetonitrile as MM region. The energy gap ΔE_{ST} was then calculated as

$$\Delta \mathrm{E}_{\mathrm{ST}} = \mathrm{E}_{\mathrm{singlet}} - \mathrm{E}_{\mathrm{triplet}} \quad (1)$$

where $E_{singlet}$ and $E_{triplet}$ are the average QM energies of the singlet and triplet snapshots, respectively. The QM energies of $^1Ph_2C$ and $^3Ph_2C$ as well as $\Delta \mathrm{E}_{\mathrm{ST}}$ in MeCN are given in Supplementary Table 2.

The $\Delta \mathrm{E}_{\mathrm{ST}}$ in MeCN was calculated to be 2.0 kcal/mol at the QM/MM level, which is in good agreement with the experimental $\Delta \mathrm{E}_{\mathrm{ST}}$ in MeCN (2.63 kcal/mol). The calculated gas phase $\Delta \mathrm{E}_{\mathrm{ST}}$ of Ph_2C was 5.3 kcal/mol. This indicates that $^1Ph_2C$ is stabilized by the polar MeCN solvent with respect to the gas phase. We should note that even with such stabilization, the triplet is still more stable than the singlet state.

Pure methanol

The $\Delta \mathrm{E}_{\mathrm{ST}}$ of Ph_2C in pure MeOH was calculated using the aforementioned protocol. The QM energies of singlet and triplet states of Ph_2C and $\Delta \mathrm{E}_{\mathrm{ST}}$ in methanol are given in Supplementary Table 3. The $\Delta \mathrm{E}_{\mathrm{ST}}$ in methanol was found to be -7.7 kcal/mol.

Unlike MeCN, MeOH reverses the singlet-triplet energy gap. The optimized structures reveal that MeOH forms a strong hydrogen bond with $^1Ph_2C$ through the carbene center, which acts as hydrogen bond acceptor (Supplementary Figure 3). This stabilizes the singlet state of Ph_2C. The triplet state, being less polar, is not equally stabilized by MeOH. This leads to the reversal of $\Delta \mathrm{E}_{\mathrm{ST}}$ in MeOH, compared to that in the gas phase or MeCN environment. Further analysis of the Ph_2C-MeOH complex showed that the average distance of the hydrogen bond in $^1Ph_2C$ (1.86 Å) was found to be at least 0.3 Å shorter than that for a hydrogen bonded complex of $^3Ph_2C$ (2.17 Å), corroborating that the singlet state is better stabilized in MeOH.

The singlet-triplet energy gap in MeCN/MeOH mixtures (Supplementary Table 4) shows that $^1Ph_2C$ is more stable than $^3Ph_2C$ by 9.8 kcal/mol in the 80:20% mixture and by 9.7 kcal/mol in the 99:1% mixture. This suggests that a single hydrogen bond can reverse the energy gap relative to gas phase. It should be noted that, for the 99:1% mixture, it is less probable for MeOH to encounter Ph_2C. Therefore, the QM/MM MD simulations and subsequent optimizations were performed with an initial structure in which Ph_2C is hydrogen bonded to MeOH.

Moving from the gas phase to MeCN, both singlet and triplet states are stabilized in the presence of solvent, however to different extent. This results in the reduction of the S-T energy gap. When the solvent is or includes MeOH, the energy gap is inverted, owing to the strong hydrogen bonding with $^1Ph_2C$.

We performed 10 ns classical MD simulation of $^{1}Ph_2C$ in both MeOH and MeCN/MeOH mixtures and analyzed the occurrences of hydrogen bonding in the simulation time. The incidents of $Ph_2C^{\cdots}HOMe$ complex were found to be 19.1%, 2.8% and 0.3% for pure MeOH, 80:20% mixture and 99:1% mixture, respectively. This indicates that diffusion might play a crucial role in the case of low MeOH fractions.

We also analyzed the distribution of $E_{singlet}$ and $E_{triplet}$ during the QM/MM MD simulations (Supplementary Figure 4). The singlet and triplet energies in different solvent mixtures showed similar trends as in the QM/MM optimizations. The triplet state was more stable in MeCN and the singlet state was stabilized in the presence of MeOH. It must be noted that the comparison of singlet and triplet energies from MD simulation is only qualitative, due to the thermal fluctuations.

Supplementary Note 4: Hydrogen bonding

Hydrogen bonding in methanol

We performed series of 20 ps QM/MM MD simulations with Ph_2C as QM region (B3LYP-D3/def2-SVP) and the solvents as MM region, to check the occurrences and stability of the Ph_2C···HOMe complex. For the solvent mixtures, the MD simulations were performed in two ways, i) with initial geometries in which the Ph_2C···HOMe complex is preformed and ii) with initial geometries in which no MeOH molecule is within 5 Å of the carbene center.

In MeOH, $^1Ph_2C$ forms a stable complex, which was conserved in all five trajectories (1-5 in Supplementary Figure 5). In three out of five simulations (1-3 in Supplementary Figure 5), the initially formed $^1Ph_2C$···HOMe complex was unaffected by competing neighbor MeOH molecules. In another simulation a neighboring MeOH molecule displaced the bound MeOH. This can be seen from the C–H value (distance between the carbene center and methanol hydrogen atom) that increases at 15-18 ps (4 in Supplementary Figure 5). A similar behavior was observed in the fifth simulation (5 in Supplementary Figure 5), where the neighboring MeOH molecule displaces the bound MeOH at about 6 ps. On the other hand, $^3Ph_2C$ does not form conserved hydrogen bonds with MeOH in any of the simulations. As can be seen in Supplementary Figure 5 (6-10), the C–H distance heavily fluctuates in all the simulations of the triplet. This supports the earlier argument that $^3Ph_2C$ is not well stabilized in MeOH.

Hydrogen bonding in the 80:20% solvent mixture

The first set of these simulations was performed without any MeOH molecule within 7 Å radius from the carbene center. As shown in Supplementary Figure 6a, in three out of five simulations (trajectories 1, 2 and 4) one MeOH molecule encounters $^1Ph_2C$ and forms a stable hydrogen bonded complex within 5 ps. The second set of simulations was carried out with a preformed $^1Ph_2C$···HOMe complex. In all cases, the preformed complex is stable throughout the entire simulation time (Supplementary Figure 6b).

Hydrogen bonding in the 99:1% solvent mixture

In the case of the 99:1% mixture, no $^1Ph_2C$···HOMe complex was found if the simulations were performed without any MeOH molecules within 6 Å radius of the carbene center. However, if the simulations were carried out starting with the preformed complex, the $^1Ph_2C$···HOMe complex was conserved in all cases (Supplementary Figure 7).

Supplementary Note 5: Reactivity of $^1Ph_2C$

To investigate the reactivity of $^1Ph_2C$, QM/MM MD simulations were carried out with $^1Ph_2C$ and all MeOH molecules within 5 Å radius of the carbene center as QM region. All other solvent molecules were treated at the MM level. $^1Ph_2C$ rapidly reacted with MeOH in all five simulations. In all cases, protonation of $^1Ph_2C$ was observed as the first step in the reaction. The protonation event occurs on the 1 ps timescale in all five trajectories, forming a metastable ion-pair intermediate (Figure 2 and Supplementary Figure 8). In most cases, this intermediate combines with the methoxide ion within 50 fs, to yield the final ether product. However, in one of the simulations (3 in Supplementary Figure 8), the protonated Ph_2C is stable for 13 ps and forms the final product afterwards.

It is interesting to note that, for the 99:1% mixture, no reaction of $^1Ph_2C$ with MeOH was observed in 30 QM/MM MD simulations (each of 30 ps). In only five cases, the $^1Ph_2C\cdots HOMe$ complex was already formed in the initial structures. However, in all these simulations one MeOH molecule could be found within a radius of 10 Å from the carbene center and six MeOH molecules were present in the whole system. Hence, the diffusion of MeOH to the reaction center is a limiting factor in this case.

Therefore, we performed additional QM/MM MD simulations with varying amounts of MeOH molecules (2 to 5) in the vicinity of the carbene center. The presence of two MeOH molecules facilitated the O–H insertion reaction in all 5 trajectories. The reaction was found to occur within 5 ps and followed mechanism 2. Only one MeOH molecule was found to react with $^1Ph_2C$ while the other MeOH molecule formed a hydrogen bond with the intermediate, thereby facilitating the reaction. When a larger number of MeOH molecules (3 to 5) were placed around $^1Ph_2C$, both mechanisms (1 and 2) were observed in a manner similar to pure MeOH. This also indicates that a reaction path involving a single MeOH molecule would be disfavored.

Supplementary Note 6: Analysis of transient absorption data

Fitting procedure for the peak positions of $^1Ph_2C$ and Ph_2CH^+

The peak wavelengths of $^1Ph_2C$ and Ph_2CH^+ undergo significant shifts within the first few tens of picoseconds. To quantify this behavior, we adapt a fitting procedure, which has recently been applied by Riedle et al.[4] in a similar manner. After defining a wavelength region of interest (338 to 376 nm for $^1Ph_2C$ and 416 to 447 nm for Ph_2CH^+), a parabola is fitted to transient absorption spectra of relevant time delays. The latter have carefully been selected by picking only spectra with a clearly discernible maximum. The maximum of the fitted parabola is then defined as the peak position of the respective feature. This procedure enables a sub-nm resolution, in contrast to being restricted to the wavelength increment of approximately 1.5 nm between adjacent data points (see graphical illustration for selected transient absorption spectra in Supplementary Figure 14a).

For $^1Ph_2C$, there is an initial red-shift of the peak wavelength within the first few picoseconds, characteristic for solvation of singlet carbenes,[5] continued by a distinct blue-shift for longer delay times for solutions with lower MeOH fractions (see Supplementary Figure 14b). The blue-shift on longer time scales might originate from geometrical changes of the two rings of the carbene molecule, or slow changes in the solvent environment. Since it is also observed for the measurement in pure MeCN, we tested if it might originate from small solvent impurities, but the same behavior was observed in dried MeCN and deliberate addition of tiny water amounts lead to clear differences, similar to addition of MeOH.

For Ph_2CH^+ we exclusively find shifts towards longer wavelengths (plotted in Figure 4b of the main manuscript). The latter dynamics can be fitted by a monoexponential decay function. By shifting each $\lambda(t)$-curve such that the data point with the highest time delay is set to $\lambda(t_{max})=0$, the offset value directly reveals the deviation from the final wavelength after relaxation (Supplementary Table 6).

Transient absorption of Ph_2CN_2*

A detailed view on the transient absorption of Ph_2CN_2 in MeOH within the first few picoseconds after UV-excitation is given in Supplementary Figure 9. In this magnified representation, the short-lived Ph_2CN_2* is evidenced by a distinct absorption band at 335 nm. A monoexpontial fit of these early dynamics reveals a lifetime of the excited precursor of ≈150 fs (red curve in Supplementary Figure 9). No significant deviations from this value are found in different solvent mixtures.

Transient absorption of $^3Ph_2C$

Supplementary Figure 11 depicts the transient absorption of Ph_2CN_2 under 285 nm excitation in pure MeCN and with small admixtures of MeOH at 315 nm, as well as a normalized representation for better comparison. The upcoming absorption of $^3Ph_2C$ is fitted using a monoexponential model function. The corresponding time constants can be found in Supplementary Table 7.

Fitting procedure for the time constants of $^1Ph_2C$

When analyzing the transient behavior of $^1Ph_2C$ at its central wavelength of 355 nm, besides contributions from the coherent artifact[6,7] and the short-lived absorption assigned to the excited precursor Ph_2CN_2*, dynamics are observed which strongly depend on the solvent mixing ratio (see Supplementary Figure 12). Generally, one finds slower dynamics for higher MeCN fractions. The rising dynamics at 355 nm, which e.g. comprise contributions from solvation and vibrational cooling, cannot be described by a single exponential function. However, this is possible for the decaying part of $^1Ph_2C$. The resulting time constants for different MeCN fractions are listed in Supplementary Table 8. The corresponding fitting curves (red) are displayed in Supplementary Figure 12 within the data range which has been considered in the fitting procedure.

Fitting procedure for the time constants of Ph_2CH^+

Early dynamics including the coherent artifact[6,7] are neglected for fitting the rise and decay of Ph_2CH^+ in different solvent mixtures. The time constants resulting from a model function consisting of the sum of two exponential functions as well as a constant offset value are listed in Supplementary Table 9. The table also contains the wavelength of the pixel being selected for the fitting procedure. The latter was determined by selecting the wavelength with the highest absorption change signal within the range of the Ph_2CH^+ absorption, accounting for wavelength shifts. Owing to the decreasing signal strength of Ph_2CH^+ when increasing the percentage of MeCN in the solvent mixture (see Figure 3 of the main manuscript), the evaluation is limited to datasets up to 90% MeCN. The resulting fit curves together with the experimental data are depicted in Supplementary Figure 13.

Supplementary Note 7: $^1Ph_2C^{\cdots}HOMe$ absorption

We performed N-electron valence state perturbation theory (NEVPT2) calculations for the complete active space (CAS) reference states, to obtain the UV-vis transitions of $^1Ph_2C$ and its complexes with water and MeOH.[8] In addition to the gas-phase $^1Ph_2C^{\cdots}HOMe$ complex, QM/MM NEVPT2/CASSCF calculations were carried out for the $^1Ph_2C^{\cdots}HOMe$ complex (QM region) in explicit MeOH solvent (MM region). The CASSCF/Def2-TZVP calculations were performed using an active space of ten orbitals and ten electrons (Supplementary Figure 10). For the gas phase $^1Ph_2C$, five singlet states were averaged with equal weight for each state. For all other cases, five singlet states and one triplet state were included for state-averaging. The NEVPT2/CASSCF calculations were performed using the ORCA program (version 3.0.3).[9]

In Ref. 10, it was shown that diphenylcarbene switches its spin to the singlet state when interacting with single water molecules embedded in an argon matrix at low temperatures by forming a strong hydrogen-bonded complex. The UV-vis spectra of the $^1Ph_2C^{\cdots}HOH$ complex is characterized by a broad band centered at 360 nm (π-π* electronic transition), in close agreement with the observation of a transient band with λ_{max} = 370 nm assigned to $^1Ph_2C$ by Kohler et al.[11] and also observed in the studies of this paper (see e.g. data for pure MeCN in Figure 3, hence for $^1Ph_2C$ without any MeOH bound to it). These experimental findings already indicate that the complex formation has only a small influence on the spectral position of the electronic absorption.

The UV-vis transitions of $^1Ph_2C^{\cdots}HOH$ were also calculated at the NEVPT2/CASSCF(10,10) level (see Supplementary Figure 10, Supplementary Table 5), which predict the strongest electronic transition at 329 nm. Similarly, the spectral transitions calculated for $^1Ph_2C$ complexed with MeOH are very similar to those predicted for $^1Ph_2C$---HOH. The transitions that contribute to the excitation (at 329 nm) are σ_c->π_c, σ_c->π*, π->π_c and π->π*, where σ_c and π_c are non-bonded carbene orbitals. Interestingly, the electronic absorption spectra of uncomplexed $^1Ph_2C$ calculated by NEVPT2/CASSCF(10,10) is very similar to those calculated for both complexes, predicting the most probable transition at 349 nm. The red shift in the transitions corresponding to uncomplexed $^1Ph_2C$ is due to the fact that the non-bonded σ-electrons are not well stabilized in the absence of hydrogen bond donors (water or methanol), which slightly decreases the σ_c->π_c and σ_c->π* energy gaps. Thus, the results from the calculations and the comparison of matrix-isolation data on $^1Ph_2C^{\cdots}HOH$ and our ultrafast data are consistent with an experimental electronic absorption spectrum of uncomplexed $^1Ph_2C$ being similar to those of both $^1Ph_2C^{\cdots}HOH$ and $^1Ph_2C^{\cdots}HOMe$.

Supplementary Note 8: Evaluation of Figure 5(a)

In this section, we discuss the data evaluation leading to Figure 5a of the main manuscript allowing for a quantitative comparison between singlet molecules that either react towards the Ph_2CH^+ or form $^3Ph_2C$. Note that our analysis relies on the assumption of an equal absorption cross section of Ph_2CH^+ and $^3Ph_2C$, respectively, in all solvent mixtures. The relative amount of Ph_2CH^+ being produced in different solvent mixtures of MeOH and MeCN can be quantified by integrating the area under the fitted transients (confer Supplementary Figure 13) between 4 and 400 ps time delay neglecting the contribution of the constant offset, and multiplying the resulting values by the corresponding rate constants describing the decay of Ph_2CH^+ in the respective solvent mixture (confer rate models described in Supplementary Note 9). Since in pure MeCN no singlet carbene takes up a proton to form Ph_2CH^+, the latter values are eventually normalized between 0.3 and 0 [see green curve in Figure 5a of the main manuscript]. For comparison, we evaluate the amount of $^3Ph_2C$ being produced by assuming that in pure MeCN all singlet carbenes undergo ISC towards the triplet state, whereas none do so in MeOH, and normalize the result between zero and one [see blue curve in Figure 5a of the main manuscript]. Thereby, the triplet strength for the respective solvent mixture is directly taken from the transient absorption data: at 315 nm we find constant absorption change signals for all solvent mixtures between 1.5 ns and 4 ns, representing the GSB of the precursor for lower MeCN fractions and the triplet absorption for higher MeCN fractions. Before normalization, data points from this temporal region of interest are averaged to enhance the data quality when quantifying the amount of $^3Ph_2C$ being produced. We assume the amount of $^3Ph_2C$ to be directly proportional to the transient absorption signal at 315 nm because any contribution from the spectrally adjacent $^1Ph_2C$ absorption band has completely vanished after 1.5 ns pump-probe delay.

To avoid errors from the assumption that there is no ISC in neat MeOH, the modelled curves in Figure 5a are calculated accordingly, thus also providing a scale between 0 and 1, i.e.

$$T(x) = \frac{T(x) - T(x=0)}{T(x=1) - T(x=0)} \quad , \tag{27}$$

with the MeCN mole fraction x and the amount T of $^3Ph_2C$ molecules.

Supplementary Note 9: Modeling the experimental data with rate models

In order to analyze how many molecules follow a certain reaction path, we have determined the rate models for different scenarios and derived the amounts for each reaction channel.

Model 1: Triplet and benzhydryl cation only

If we assume that the singlet carbene $^1Ph_2C$ (for simplicity called "S" in the following equations) can either react to the triplet $^3Ph_2C$ ("T"), as in pure MeCN, or to the benzhydryl cation Ph_2CH^+ ("B"), as seen in MeOH, which further turns into the ether product ("E"), we can derive the following rate equations

$$[\dot{S}] = -k_{SB}[S] - k_{ST}[S] \quad (2)$$

$$[\dot{T}] = +k_{ST}[S] \quad (3)$$

$$[\dot{B}] = +k_{SB}[S] - k_{BE}[B] \quad (4)$$

$$[\dot{E}] = +k_{BE}[B] \quad (5)$$

$$[S]_{t=0} = S_0;\ [T]_{t=0} = 0;\ [B]_{t=0} = 0; [E]_{t=0} = 0 \quad (6)$$

which have as solution:

$$[S] = S_0 e^{-(k_{SB}+k_{ST})t} \quad (7)$$

$$[T] = S_0 \frac{k_{ST}}{k_{SB}+k_{ST}}[1 - e^{-(k_{SB}+k_{ST})t}] \quad (8)$$

$$[B] = S_0 \frac{k_{SB}}{k_{SB}+k_{ST}-k_{BE}}[e^{-k_{BE}t} - e^{-(k_{SB}+k_{ST})t}] \quad (9)$$

$$[E] = S_0\{\frac{k_{SB}}{k_{SB}+k_{ST}} + \frac{k_{SB}}{(k_{SB}+k_{ST})(k_{SB}+k_{ST}-k_{BE})}[k_{BE}e^{-(k_{SB}+k_{ST})t} - (k_{SB} + k_{ST})e^{-k_{BE}t}]\} \quad (10)$$

The amount of molecules following each reaction path are:

$$[T]_{t=\infty} = S_0 \frac{k_{ST}}{k_{SB}+k_{ST}} \quad (11)$$

$$[E]_{t=\infty} = S_0 \frac{k_{SB}}{k_{SB}+k_{ST}} \quad (12)$$

The final ether product does not show any absorption signal in the probed wavelength region. Hence, the amount of molecules following the reaction path towards the ether has to be determined from the absorption signal of the benzhydryl cation. If the experimental transient absorption is measured, this can be done by performing the integral

$$\int_0^\infty [B]dt = S_0 \frac{k_{SB}}{k_{BE}(k_{SB}+k_{ST})} = \frac{1}{k_{BE}}[E]_{t=\infty} \quad (13)$$

and subsequent multiplication of the obtained value with the experimentally determined decay rate k_{BE} of the benzhydryl cation, thus yielding $[E]_{t=\infty}$.

The rate constants in the general scheme described above can depend on the concentration of the solvent as well, i.e., $k_{SB}=k'_{SB}[M]$ should depend on the concentration of MeOH ("M"). Since the amount of MeOH is much higher than the amount of carbene already for small

mole fractions of MeOH in the binary solvent mixture, a pseudo-first-order behavior is assumed, i.e., [M] does not change during the reaction.

For an exemplary calculation, k_{ST} is determined by combining the solvent polarity dependence of k_{ST} [12] with the solvent polarity parameter $E_T(30)$ of binary mixtures of MeCN and MeOH,[13] whereas the other two rate constants are determined from experiments in neat MeOH, i.e., in neat MeOH we have k_{SB}=(12 ps)$^{-1}$ and k_{BE}=(23 ps)$^{-1}$, while in mixtures they depend on the MeOH concentration and become k_{SB}=(12 ps)$^{-1}$ [M]/[M_0]=(12 ps)$^{-1}$ v_M and k_{BE}=(23 ps)$^{-1}$ [M]/[M_0]=(23 ps)$^{-1}$ v_M, respectively, with the volume fraction v_M of MeOH and M_0 being the concentration of neat MeOH.

The result is shown in Supplementary Figure 15a. The curve for the triplet pathway resembles the experimentally determined one, whereas the other one does not. Clearly, in this *model 1* there are only two reaction paths, hence the two curves have to add up to one. Possible reasons for the discrepancy might be:

- the *model 1* on which the modeling is based is incomplete
- the assumption in the modeling that the rates are constant or only depend linearly on [M] is too simple
- the absorption cross section of Ph_2CH^+ might change drastically with the mole fraction of MeOH, so that the shape of the curve derived from the experiments is obscured – this issue has been addressed for pure MeOH and MeCN by Kohler and coworkers[11] who concluded that due to similar dielectric properties, the absorption coefficient in both solvents should be roughly the same
- since the transient absorption bands shift spectrally with time, the integration of the experimental Ph_2CH^+ signal is too inaccurate

While with all of these reasons one might rationalize that the experimentally determined amounts do not add up to one, the observation that the decay time of $^1Ph_2C$ and the rise time of Ph_2CH^+ differ strongly for certain solvent mixing ratios points to the first one listed (without necessarily excluding the other ones which might additionally have to be considered, see below), i.e., that *model 1* is incomplete. We therefore look for alternatives.

Model 2: A third decay channel

In the work by Kohler and coworkers,[11] it was found that the protonation fraction of all excited molecules is about 30% in neat MeOH. One possibility is that 70% of the excited Ph_2CN_2* molecules do not dissociate but relax back to the ground state of the diazo compound (as it is for example observed when exciting diazo-Meldrum's acid[14]). However, in this scenario all the generated $^1Ph_2C$ molecules will follow the protonation pathway via Ph_2CH^+. Thus, when MeCN is added, allowing also the triplet pathway, the amount of molecules following each pathway should look similar as in mechanism 1 of Figure 2. Since this is not the case, we conclude that another possibility mentioned in Ref. 11 is more appropriate, namely that 70% follow a reaction path from singlet carbene to the ether product which does not involve the Ph_2CH^+ intermediate.

The third reaction channel comprises an intermediate complex $^1Ph_2C$···HOMe ("C") of a singlet carbene and a MeOH molecule, as e.g. suggested by Eisenthal and coworkers[15] and

also observed in our simulations. From there, the ether product can be generated in a concerted fashion.

This can be expressed in the following rate equations

$$[\dot{S}] = -k_{SB}[S] - k_{ST}[S] - k_{SC}[S] \tag{14}$$

$$[\dot{T}] = +k_{ST}[S] \tag{15}$$

$$[\dot{B}] = +k_{SB}[S] - k_{BE}[B] \tag{16}$$

$$[\dot{C}] = +k_{SC}[S] - k_{CE}[C] \tag{17}$$

$$[\dot{E}] = +k_{BE}[B] + k_{CE}[C] \tag{18}$$

$$[S]_{t=0} = S_0;\ [T]_{t=0} = 0;\ [B]_{t=0} = 0;\ [C]_{t=0} = 0; [E]_{t=0} = 0 \tag{19}$$

with the solutions

$$[S] = S_0 e^{-(k_{SB}+k_{ST}+k_{SC})t} \tag{20}$$

$$[T] = S_0 \frac{k_{ST}}{k_{SB}+k_{ST}k_{SC}} [1 - e^{-(k_{SB}+k_{ST}+k_{SC})t}] \tag{21}$$

$$[B] = S_0 \frac{k_{SB}}{k_{SB}+k_{ST}+k_{SC}-k_{BE}} [e^{-k_{BE}t} - e^{-(k_{SB}+k_{ST}+k_{SC})t}] \tag{22}$$

$$[C] = S_0 \frac{k_{SC}}{k_{SB}+k_{ST}+k_{SC}-k_{CE}} [e^{-k_{CE}t} - e^{-(k_{SB}+k_{ST}+k_{SC})t}] \tag{23}$$

$$[E] = \frac{S_0}{k_{SB}+k_{ST}+k_{SC}} \{k_{SB} + k_{SC} + \left[\frac{k_{SB}k_{BE}}{k_{SB}+k_{ST}+k_{SC}-k_{BE}} + \frac{k_{SC}k_{CE}}{k_{SB}+k_{ST}+k_{SC}-k_{CE}}\right] e^{-(k_{SB}+k_{ST}+k_{SC})t} - (k_{SB} + k_{ST} + k_{SC}) \left[\frac{k_{SB}}{k_{SB}+k_{ST}+k_{SC}-k_{BE}} e^{-k_{BE}t} + \frac{k_{SC}}{k_{SB}+k_{ST}+k_{SC}-k_{CE}} e^{-k_{CE}t}\right]\} \tag{24}$$

Since we have three parallel pathways starting from $^1Ph_2C$, the amount of molecules following each path are simply:

$$[T]_{t=\infty} = S_0 \frac{k_{ST}}{k_{SB}+k_{ST}+k_{SC}} \tag{25}$$

$$[E]_{t=\infty} = S_0 \frac{k_{SB}}{k_{SB}+k_{ST}+k_{SC}} + S_0 \frac{k_{SC}}{k_{SB}+k_{ST}+k_{SC}} \tag{26}$$

where equation (26) is a sum of the paths involving either Ph_2CH^+ or the complex $^1Ph_2C^{\cdots}HOMe$.

Both for reaching Ph_2CH^+ and $^1Ph_2C^{\cdots}HOMe$, the singlet carbene has to interact with a MeOH molecule. First, we will assume that the rates are pseudo-first order with a linear dependence on [M], i.e., $k_{SB}=k'_{SB}[M]$ and $k_{SC}=k'_{SC}[M]$. In order to take into account that only ≈30% of singlet molecules follow the Ph_2CH^+ pathway and that Ph_2CH^+ in neat MeOH rises with a time constant of 12 ps, we use $k'_{SB}=(40\text{ ps})^{-1}/M_0$ and $k'_{SC}=(17.6\text{ ps})^{-1}/M_0$ in the calculation.

The result is shown in Supplementary Figure 15b. As expected, the shape of the curves has not changed, but now only 30% proceed via the cation towards the ether product. With the assumptions outlined above, the model gives no indication why the amount of molecules

following the $^1Ph_2C \rightarrow Ph_2CH^+ \rightarrow$ ether pathway shows an almost linear dependence on the concentration.

Model 3: non-linear dependence on methanol concentration

Instead of including further pathways or equilibria, we concentrate on the dependence on [M]. Especially, we are interested in the question what governs whether there will be protonation or a complexation in the interaction of $^1Ph_2C$ with MeOH. Kirmse and Steenken[16,17] suggested that two MeOH molecules are needed for the protonation, our simulations (mechanism 1 in Figure 2 of the main manuscript) reveal the involvement of at least two MeOH molecules as well. In addition, Scaiano and coworkers,[18] for other carbenes in MeCN with little amounts of MeOH, have observed that MeOH oligomers are more reactive than monomers.

We consider MeOH dimers being present in the solution, with the associated equilibrium 2 MeOH$\leftrightarrow$(MeOH)$_2$ with equilibrium constant K. These might originate from hydrogen bonding of two adjacent MeOH molecules. For the Ph_2CH^+ formation, such a dimer would serve two processes: First, the presence of the hydrogen bond can facilitate the MeOH molecule's ability to donate a proton. Second, when MeOH transfers a proton to $^1Ph_2C$ to yield Ph_2CH^+, a methoxide anion is created as well which could geminately recombine with the cation. This process might be efficiently avoided if the bonded MeOH transfers a proton to the methoxide, itself turning into a methoxide anion which however is further away from the cation.

Therefore, we modify the rate constant k_{SB}, which depends on the dimer concentration and hence $k_{SB}=k''_{SB}[(MeOH)_2]=k''_{SB}K[M]^2$. For the modeling, we use $k'_{SB}K=(40\text{ ps})^{-1}/M_0^2$ to match the experimental rates in neat MeOH. The result is shown in Supplementary Figure 15c. The curve for the reaction path involving the cation is now close to a linear behavior.

Decay of the singlet absorption

Our experimental data (confer Figure 4a of the main manuscript) shows that the decay rate of the singlet absorption signal is always lower than the rate associated with the rise of the benzhydryl cation. This can be explained if the complex $^1Ph_2C$···HOMe has absorption characteristics in the visible spectral domain which are very similar to those of $^1Ph_2C$. Then, the observed singlet absorption also originates from the complex. In the complex, the MeOH is bound to $^1Ph_2C$ and can eventually react with it in a concerted fashion to form the ether product. The lifetime of this complex will strongly depend on the environment, e.g. because the solvent polarity and the possibility for hydrogen bonding to another MeOH will change. To model the decay, we assume that the rate k_{CE} is also pseudo-first order, i.e., $k_{CE}=k'_{CE}[M]$, with a rate on the order of diffusion $k'_{CE}=(18\text{ ps})^{-1}/M_0$. This linear dependence is also motivated by mechanism 2 found in the simulations (Figure 2 in the main manuscript).

Supplementary Figure 16 shows exemplary transients which result from this model. Note in the right panel that the initial rise of the singlet carbene absorption signal which was observed in the experiments can be explained if the absorption coefficient for the complex $^1Ph_2C$···HOMe is larger than that of $^1Ph_2C$. Further note that the rising dynamics are also observed in pure MeCN and might originate from solvation and vibrational cooling, possibly

slight solvent impurities, or a slower buildup of the dipole moment in the complex, e.g. because of geometrical changes.

The decay rate of the absorption signal of Ph_2CH^+ (see Figure 4a in the main manuscript) exhibits a basically linear dependence on [M] as well. Kohler and coworkers[11] deduced by variation of the alcohol that the Ph_2CH^+ cation reacts with a further neutral alcohol molecule rather than the alkoxide. Modeling the transient absorption signals of Ph_2CH^+ with a decay rate of $k_{BE}=k'_{BE}[M]=(23\ ps)^{-1}[M]/M_0$, derived from the decay curve of the Ph_2CH^+ absorption in neat MeOH, yields curves which qualitatively reproduce the experimental ones.

Rate constants

Within the model sketched in Figure 5b of the main manuscript, we can also calculate the rates associated with the rise and decay of the Ph_2CH^+ absorption signal. These are shown in Supplementary Figure 17. Although the quadratic dependence on [M] drastically changes how many molecules take a certain path, the rate constant for Ph_2CH^+ formation (black) is still almost linear, since it is governed by the parallel singlet decay channel towards the complex $^1Ph_2C{\cdots}HOMe$. The complex rises with the same rate constant with which the singlet decays via three parallel channels. We further see that the decay constant of the complex is always smaller than the one of the Ph_2CH^+ rise, explaining why in the experiment the combined absorption of $^1Ph_2C$ and $^1Ph_2C{\cdots}HOMe$ decays more slowly than Ph_2CH^+ appears. However, the experimental signal is the sum of several exponentials [basically of equations (20) and (23)] and does not decay monoexponentially, but if $^1Ph_2C$ decays much faster than $^1Ph_2C{\cdots}HOMe$, then the decay rate is approximately the one of the complex.

The experimentally determined rate constants also show a behavior which is almost linear with respect to the mole fraction (Figure 4a of the main manuscript). The most pronounced deviation is for the rise of Ph_2CH^+ in low MeCN concentrations. This might indicate that 90% or 100% MeOH does not make a big difference anymore for the rate constant, because there are always several MeOH molecules close-by.

Supplementary Note 10: Power-dependence of transient absorption spectra

Care was taken to ensure that the series of transient absorption measurements on Ph_2CN_2 in various solvent mixtures of MeOH and MeCN was carried out in the linear excitation regime. To guarantee this, we recorded transient absorption spectra for different excitation energies. Owing to our experimental setup, the latter can be controlled by the adjustable output of the Dazzler pulse shaper. The resulting pulse energy was measured before the sample position. Data for an exemplarily chosen solvent mixture at a certain time delay is depicted in Supplementary Figure 18a. When plotting the resulting absorption change signals for any wavelength (Supplementary Figure 18b, data is exemplarily shown for a probe wavelength of 360 nm), one finds linear relationships, which at least hold for excitation energies up to 134 nJ, constituting the upper limit of excitation power available in the given experimental setup. In the actual measurement series, an excitation power of 130 nJ was used to achieve an excellent signal-to-noise ratio.

SUPPLEMENTARY REFERENCES

1. Eisenthal, K. B., Moss, R. A. & Turro, N. J. Divalent Carbon Intermediates: Laser Photolysis and Spectroscopy. *Science* **225,** 1439–1445 (1984).
2. Hess, B., Kutzner, C., van der Spoel, D. & Lindahl, E. GROMACS 4: Algorithms for Highly Efficient, Load-Balanced, and Scalable Molecular Simulation. *J Chem Theor Comput* **4,** 435–447 (2008).
3. Marcus, Y. Preferential solvation in mixed solvents. Part 6. Binary mixtures containing methanol, ethanol, acetone or triethylamine and another organic solvent. *J Chem Soc Faraday Trans* **87,** 1843 (1991).
4. Sailer, C. F. *et al.* A Comprehensive Microscopic Picture of the Benzhydryl Radical and Cation Photogeneration and Interconversion through Electron Transfer. *ChemPhysChem* **14,** 1423–1437 (2013).
5. Wang, J., Kubicki, J., Gustafson, T. L. & Platz, M. S. The Dynamics of Carbene Solvation: An Ultrafast Study of p-Biphenylyltrifluoromethylcarbene. *J Am Chem Soc* **130,** 2304–2313 (2008).
6. Megerle, U., Pugliesi, I., Schriever, C., Sailer, C. F. & Riedle, E. Sub-50 fs broadband absorption spectroscopy with tunable excitation: putting the analysis of ultrafast molecular dynamics on solid ground. *Appl Phys B* **96,** 215–231 (2009).
7. Ekvall, K. *et al.* Cross phase modulation artifact in liquid phase transient absorption spectroscopy. *J Appl Phys* **87,** 2340 (2000).
8. Schapiro, I., Sivalingam, K. & Neese, F. Assessment of n-Electron Valence State Perturbation Theory for Vertical Excitation Energies. *J Chem Theory Comput* **9,** 3567–3580 (2013).
9. Neese, F. The ORCA program system. *WIREs Comput Mol Sci* **2,** 73–78 (2012).
10. Costa, P., Fernandez-Oliva, M., Sanchez-Garcia, E. & Sander, W. The Highly Reactive Benzhydryl Cation Isolated and Stabilized in Water Ice. *J Am Chem Soc* **136,** 15625–15630 (2014).
11. Peon, J., Polshakov, D. & Kohler, B. Solvent Reorganization Controls the Rate of Proton Transfer from Neat Alcohol Solvents to Singlet Diphenylcarbene. *J Am Chem Soc* **124,** 6428–6438 (2002).
12. Sitzmann, E. V., Langan, J. G., Griller, D. & Eisenthal, K. B. Effects of solvent polarity and structure on intersystem crossing in diphenylcarbenes. A picosecond laser study on dimesitylcarbene. *Chem Phys Lett* **161,** 353–360 (1989).
13. Ortega, J., Rafols, C., Bosch, E. & Roses, M. Solute-solvent and solvent-solvent interactions in binary solvent mixtures. Part 3. The $E_T(30)$ polarity of binary mixtures of hydroxylic solvents. *J Chem Soc Perkins Trans 2* 1497 (1996).
14. Rudolf, P., Buback, J., Aulbach, J., Nuernberger, P. & Brixner, T. Ultrafast Multisequential Photochemistry of 5-Diazo Meldrum's Acid. *J Am Chem Soc* **132,** 15213–15222 (2010).
15. Sitzmann, E. V., Langan, J. G. & Eisenthal, K. B. Picosecond laser studies of the effects of reactants on intramolecular energy relaxation of diphenylcarbene: Reaction of diphenylcarbene with alcohols. *Chem Phys Lett* **112,** 111–116 (1984).
16. Kirmse, W., Guth, M. & Steenken, S. Production of α-Siloxycarbenium Ions by Protonation of Photochemically Generated α-Siloxycarbenes. Formation Mechanism and Reactivities with Nucleophiles. *J Am Chem Soc* **118,** 10838–10849 (1996).
17. Steenken, S. Production of carbenium ions from carbenes by protonation. *Pure Appl Chem* **70,** (1998).
18. Griller, D., Nazran, A. S. & Scaiano, J. C. Reaction of diphenylcarbene with methanol. *J Am Chem Soc* **106,** 198–202 (1984).
